# An Overview of Degradation Strategies for Amitriptyline

**DOI:** 10.3390/ijms25073822

**Published:** 2024-03-29

**Authors:** Cezar Comanescu, Radu C. Racovita

**Affiliations:** 1 Department of Inorganic Chemistry, Physical Chemistry and Electrochemistry, Faculty of Chemical Engineering and Biotechnologies, National University of Science and Technology POLITEHNICA Bucharest, 1-7 Gh. Polizu St., District 1, 011061 Bucharest, Romania; 2 National Institute of Materials Physics, Atomistilor 405A, 077125 Magurele, Romania; 3 Faculty of Physics, University of Bucharest, Atomistilor 405, 077125 Magurele, Romania

**Keywords:** antidepressant drugs, amitriptyline, drug stability, degradation pathways, chemical degradation, oxidation, hydrolysis, photodegradation/photostability, forced degradation, quality control, shelf life

## Abstract

Antidepressant drugs play a crucial role in the treatment of mental health disorders, but their efficacy and safety can be compromised by drug degradation. Recent reports point to several drugs found in concentrations ranging from the limit of detection (LOD) to hundreds of ng/L in wastewater plants around the globe; hence, antidepressants can be considered emerging pollutants with potential consequences for human health and wellbeing. Understanding and implementing effective degradation strategies are essential not only to ensure the stability and potency of these medications but also for their safe disposal in line with current environment remediation goals. This review provides an overview of degradation pathways for amitriptyline, a typical tricyclic antidepressant drug, by exploring chemical routes such as oxidation, hydrolysis, and photodegradation. Connex issues such as stability-enhancing approaches through formulation and packaging considerations, regulatory guidelines, and quality control measures are also briefly noted. Specific case studies of amitriptyline degradation pathways forecast the future perspectives and challenges in this field, helping researchers and pharmaceutical manufacturers to provide guidelines for the most effective degradation pathways employed for minimal environmental impact.

## 1. Introduction

In a society where stressors can be found all around, the prevalence of mental disorders reached, according to a 2019 report from the WHO (World Health Organization), one in every eight people (roughly 12.5% of the world population) [1], with the potential to severely impact the life quality of those affected [2,3,4,5,6,7,8,9,10,11,12,13,14,15,16,17,18,19,20,21,22,23,24,25,26]. In other words, ~1 billion people suffer from some form of mental health condition. During the COVID-19 pandemic, anxiety and depressive disorders rose to a whopping 28% [4,5,6,7,8,9,19,23,27,28,29,30,31,32]. There are many types of mental disorders that can cause disturbances in cognition, behavior, or emotional regulation [6,14,15]. Although prevention and treatment options are more diversified today than ever, only a few of those affected have access to effective care (only one in every three people dealing with depression according to the same WHO report from 2019) while also being at risk of experiencing stigma or discrimination. When properly diagnosed, antidepressants are the go-to drugs, providing key medical treatment options with high importance in the management of mental health disorders. Antidepressants have revolutionized the field of mental health treatment, offering effective pharmacological interventions for various mental health disorders. The study of degradation strategies for antidepressant drugs stems, on one hand, from the need to ensure more efficient treatment for those in need without significant degradation and, on the other hand, from the need to safely dispose of residual antidepressant drugs from water bodies [19,25,33,34,35,36].

This review provides an up-to-date overview of degradation strategies for amitriptyline, one of the most prescribed TCAs, which will guide its further removal from the environment in a safe, reliable, and sustainable manner. By exploring current degradation options, researchers can opt for specific reagents or conditions aimed at reducing environmental pollution through the degradation of antidepressants and/or their degradation products. Antidepressants have a crucial role in the treatment of mental health disorders; thus, ensuring the stability and quality of these medications [4] is of utmost importance to facilitate their therapeutic efficacy and patient safety [2,37,38,39,40,41]. Considering the various chemical, physical, and environmental factors can lead to valuable insights into ensuring the stability, efficacy, and safety of antidepressant medications, as well as the most environmentally benign routes to their degradation [42].

Recent years have witnessed rapid progress in formulation technologies, such as nanotechnology and prodrug approaches [15,41,43,44,45]. These advancements offer innovative strategies for enhancing drug stability, controlled release, and targeted delivery, ultimately improving therapeutic outcomes for patients. The growing importance of green chemistry and sustainable approaches in drug development is also acknowledged [27,39,46,47,48,49]. The integration of environmentally friendly manufacturing processes and eco-friendly packaging materials contributes to reducing the environmental footprint of the pharmaceutical industry while maintaining the stability and quality of antidepressants [50,51,52,53,54,55].

Personalized medicine emerges as a promising approach to addressing stability concerns. The ability to tailor drug stability to individual patient needs through pharmacogenomics and customized formulations minimizes the risk of degradation-related therapeutic failures. The significance of quality control and regulatory considerations in ensuring the stability of antidepressant drugs must be emphasized. Harmonization of stability guidelines across regulatory agencies, strategies for addressing stability challenges associated with complex drug products and combination therapies, and robust post-market surveillance are crucial in maintaining the stability and integrity of these medications [32,41,56,57,58,59,60,61,62,63].

There are several challenges related to analytical techniques and method development aimed at detecting trace degradation products and impurities and at developing stability-indicating assays [56]. Ongoing research, collaboration, and innovation seek to address antidepressant drug degradation, ensuring the availability of stable and effective antidepressant therapies, as well as minimal risks of toxicity to the environment [15,22,41,64,65,66,67,68].

## 2. Overview of Antidepressant Drug Degradation

Mental health disorders are a current threat to the wellbeing of today’s society, and recent reports point out the alarmingly early onset of various diseases that were initially thought to affect only the elders; for instance, a report from 2023 identifies the earliest patient confirmed with Alzheimer’s disease to be a young individual at the age of 19. With an increasing prevalence (one in eight people suffer from a mental disorder according to the WHO), mental health issues need to be better handled for the generations to come. Antidepressant drugs are a current treatment option for patients suffering from mental health disorders [14]. With increasing prescriptions of antidepressants, an increasing concern is now rising regarding the environmental impact of antidepressants disposed of in water bodies throughout the globe [21,25,60,67]. As first-line treatment options for mental health disorders, their stability and integrity are essential for maximum efficiency; however, the therapeutic properties of antidepressants can be compromised due to various degradation pathways, including thermal degradation, hydrolysis (forced hydrolysis, extreme pH hydrolysis), oxidation, and photodegradation [69,70]. Understanding the details of degradation processes is essential for ensuring the efficacy, safety, and shelf life of these medications [35,71,72,73].

### 2.1. Drug Degradation and Its Implications

**Figure 1:**
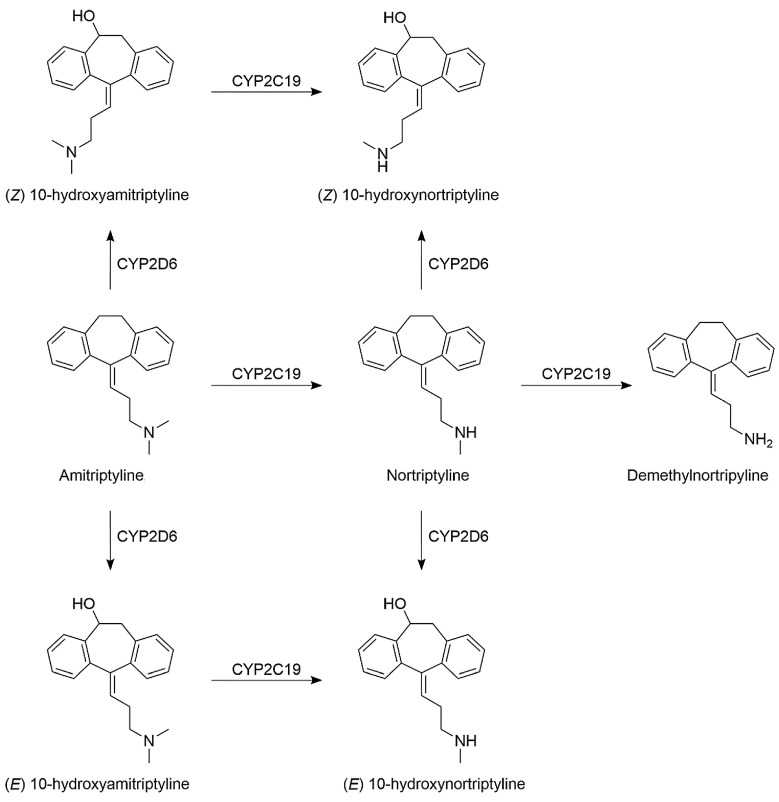
Metabolism of amitriptyline by P450 enzymes. Reproduced with permission from [74].

There are significant implications that drug degradation can have for the efficacy, safety, and overall quality of antidepressant medications, as well as lowering the quality of environmental parameters, urging researchers to identify and scale up degradation procedures. One of the primary implications of drug degradation is the reduced therapeutic effectiveness of antidepressant drugs. As the second TCA introduced in 1961 for the treatment of major depression disorder (MDD), amitriptyline has also raised concerns over its potential cardiotoxicity, which eventually led to the discontinuation of Elavil tablets in 2017 [74]. With a low toxic dose of 5 mg/kg, amitriptyline can be administered in therapeutic daily doses up to 150 mg, regardless of patient weight, and it accounts for about 40% of fatalities (suicide) related to antidepressant overdose in the USA [74]. Degradation reactions can lead to the breakdown of active pharmaceutical ingredients (APIs) into inactive or less potent forms, resulting in a decrease in the drug’s ability to elicit the desired therapeutic response. This can lead to treatment failure or inadequate symptom relief in patients, negatively impacting their mental health outcomes. AMT can be metabolized by the P450 hepatic enzymes CYP2C19 (demethylation of AMT to nortriptyline (major metabolite) and further to demethylnortriptyline) and CYP2D6 (hydroxylation of nortriptyline to (Z)-10-hydroxynortriptyline and (E)-10-hydroxynortriptyline in a 1:3 ratio) (Figure 1) [74].

Moreover, the formation of degradation products during storage or usage may introduce potentially harmful impurities with potential safety risks to patients, as they may exhibit unexpected toxicity or side effects [37,38]. Proper identification and characterization of degradation products remain critical for assessing their impact on patient safety. When overdosed, AMT can lead to sudden cardiac death due to sarcoplasmic inhibition of calcium reuptake and through the inhibition of the hERG potassium channel [74]. Other side effects should be accounted for, such as the sedative effect of AMT in low doses, which could also be responsible for weight gain observed in many patients due to increased basal appetite.

Another implication of drug degradation is the risk of altered pharmacokinetics [75]. Degradation processes can affect a drug’s stability, solubility, and dissolution rate, leading to variations in drug absorption and bioavailability. Attention should be paid by patients not to associate AMT with alcohol or other central nervous system depressants, as this can enhance their effects [74]. Drug degradation can also raise regulatory concerns, as regulatory authorities impose strict guidelines on drug stability and quality [58,68,69,73,76]. Addressing drug degradation implications requires comprehensive stability studies during drug development and post-marketing surveillance [43,77,78]. In a recent study conducted in Western Cape, South Africa, it was found that 6.5% of the elderly patients were inappropriately prescribed amitriptyline, which posed real risks to the exposed patients, especially those with co-morbid cardiovascular conditions [79]. Depending on the nature of the condition that it addresses, AMT could be prescribed for the treatment of pain, sleep disorders, or physical fatigue, and proper understanding of each mechanism of action could provide a valuable pharmacological profile for future drug development [80].

### 2.2. Factors Influencing Drug Degradation

Different strategies have been employed in drug degradation processes, such as photodegradation, bacterial degradation, and catalytic degradation with inorganic or organometallic catalysts [42]. The key feature guiding proper degradation strategies lies in the chemical structure of the drug molecule itself. Typically, antidepressants contain various functional groups (such as esters, amides, and carboxyl) and double bonds that are susceptible to hydrolysis or oxidation. The presence of these labile functional groups renders the drug more prone to degradation under specific conditions; for instance, a C=C bond will likely undergo oxidation when treated with an oxidation reagent. Additionally, the drug’s overall chemical stability, including the presence of stabilizing groups or conjugated systems, can influence its susceptibility to degradation reactions [67].

The factors contributing to AMT degradation have been investigated in the past given the long history of AMT on the market [81]. Evener et al. showed in 1977 that AMT hydrochloride was degraded in a buffered solution by a chemical oxidation pathway, which was faster when traces of metal ions were present as contaminants (Cu(OAc)_2_ or FeCl_3_) [81]. This accelerated decomposition observed under metal ion catalysis could be mitigated by the addition of disodium salt of EDTA, which is known for its strong chelating effect on cations, confirming that metallic impurities likely leaching from containers are a real source of concern [81]. Under those experimental conditions, Na_2_S_2_O_5_ (sodium metabisulfite) was found responsible for the accelerated oxidation of AMT through the attack of pyrosulfite at the C=C olefinic bond of AMT in a free radical mechanism following second-order kinetics and leading to dibenzocycloheptanone as the only decomposition product [81].

Environmental conditions (temperature [82], humidity, and light exposure) can make a drug prone to degradation. Elevated temperatures can accelerate chemical reactions, leading to faster degradation rates. Similarly, exposure to high humidity can promote hydrolytic degradation. Light exposure, particularly exposure to UV and visible light, can initiate photochemical reactions, causing photodegradation of susceptible drug molecules [36,83,84]. As a result, proper storage and transportation conditions are essential to preserving drug stability. The pH of a drug’s environment can alter its bioavailability while also affecting its degradation rate. Hydrolysis is highly dependent on the pH of the medium, with ester hydrolysis being more pronounced in acidic conditions and amide hydrolysis being favored under alkaline conditions [85]. Understanding the pH-dependent reactivity of drug functional groups is critical for designing stable drug formulations that maintain efficacy under various physiological conditions. A valuable indicator of AMT degradation is the half-life in freshwater sediments, with a long period of 78.8 d being noted in the case of AMT [86].

The presence of impurities in a drug formulation may even accelerate its degradation [32]. Even trace amounts of catalysts, metal ions, or other contaminants can catalyze degradation reactions, leading to the formation of degradation products and prompting rigorous quality control and purification procedures [81]. The addition of antioxidants such as propyl gallate (and, to a lesser extent, hydroquinone) managed to stabilize an AMT solution, albeit at the cost of a 10% initial concentration decrease [81]. Formulation and excipient selection are also key factors influencing drug stability and shelf life. The choice of excipients and drug–excipient compatibility can impact drug solubility, dissolution rate, and chemical environment, affecting the susceptibility to degradation [54,87].

### 2.3. Degradation Pathways for Antidepressant Drugs

Various approaches and techniques have been explored to control and manage the chemical degradation of antidepressant drugs, with the main mechanisms being hydrolysis, oxidation, photodegradation, and temperature-induced reactivity. Environmental considerations are vital in managing the chemical degradation of antidepressant drugs when released into the environment. Advanced wastewater treatment technologies can help remove or reduce pharmaceutical residues from wastewater effluents, limiting the potential for environmental exposure and ecological impacts [28,64]. Additionally, improved pharmaceutical disposal practices and public awareness programs can prevent the improper disposal of drugs, which can contribute to environmental contamination [2].

Hydrolysis can cleave ester, amide, or other labile functional groups present in a drug’s structure. Hydrolytic degradation is often influenced by factors such as pH, temperature, and the presence of catalysts, making it a significant concern for drug stability during storage and usage [7,19,71,75]. Forced degradation and stability assessment tools [62,71,73,77,87,88] may shed light on the potential toxicity of the decomposition products. Scientists have identified various chemical degradation pathways that antidepressant medications may undergo, with potential loss of their efficacy. This can help researchers design more stable and effective drug formulations [72,89,90].

Conducting early screening enables the identification of drug candidates that exhibit higher chemical stability, reducing the risk of degradation during storage and use [29,33,90]. Structural modifications can often occur by altering specific functional groups that are susceptible to various types of degradation. Computational studies are of great importance, aiding and complementing experimental results [19]. Formulation optimization may include appropriate excipients, such as antioxidants, chelating agents, stabilizers, protective coatings, or encapsulation techniques to shield the drug from light exposure, minimizing photodegradation [2,73].

Thermal degradation is a potential tool for degrading antidepressants. Depending on the functional groups contained and the molecular complexity of the drug, some TCAs may degrade faster than others. For instance, IMI (R–N(CH_3_)_2_) degrades around 150 °C, which is faster than DES (R–NH(CH_3_); degradation ~170 °C) due to the increased substitution degree of the amine nitrogen in the case of IMI. However, the temperature range for thermal decomposition exceeds 100 °C; therefore, this degradation could be useful for solid-state medicine rather than aqueous solutions thereof. Roman et al. conducted a stability study of AMT hydrochloride solution when stored at 80 °C for 3 months, noting a 1.3% degradation over the course of 90 days (10.08 mg/mL after 3 months vs. 10.21 mg/mL initially) [82]. On the other hand, neat AMT exhibits a rather high thermal stability (up to 188 °C [11]).

Oxidation is a major degradation pathway for antidepressants and TCAs in particular [8]. Oxidative degradation can be catalyzed by light, metal ions, or other species, leading to the generation of reactive oxygen species. This process mainly exposes unsaturated bonds, which are prone to undergoing oxidation by many mild or strong oxidants [4,8,27].

Photodegradation is a crucial pathway for antidepressant drugs that are sensitive to exposure to ultraviolet or visible light [9,19,91,92]. This degradation can be significant for drugs intended for oral or transdermal administration, where exposure to sunlight during storage or use can be a concern [78,93]. Additionally, specific drug–drug interactions may accelerate degradation, especially when antidepressants are co-administered with other medications. Proper knowledge of degradation pathways for antidepressants can guide the development of suitable storage conditions and packaging materials shielding the drugs from environmental factors inducing degradation [72,94].

### 2.4. Monitoring and Analytical Techniques

Monitoring the chemical stability of antidepressant drugs can be achieved by means of analytical techniques such as chromatography, spectroscopy, and mass spectrometry [95]. For instance, a quality by design (QbD) approach was inferred in the case of AMT and its four main impurities [95]. Continuous monitoring and analysis enable timely intervention and formulation improvements to enhance the stability of antidepressants during storage, transportation, and usage [87]. Stability studies are conducted under controlled conditions of temperature, humidity, and light to assess the drug’s stability over time. Accelerated stability studies involve subjecting the drug to elevated stress conditions to predict its long-term stability within a shorter time frame, providing valuable information on the drug’s degradation kinetics [72].

High-performance liquid chromatography (HPLC) is a widely used analytical technique for monitoring drug degradation, allowing the separation and quantification of drug compounds, degradation products, and potential impurities. By analyzing drug samples at various time points during stability studies, researchers can identify degradation pathways, detect any changes in drug content, and assess drug purity [37,77]. Mass spectrometry (MS) can be coupled with HPLC to enable the identification and structural characterization of drug degradation products and impurities. By providing information about the molecular weight, possible fragmentation patterns, and chemical properties of the detected species, MS is a key method for addressing potential toxicity concerns [37]. Spectroscopic methods, such as infrared spectroscopy (FT-IR) and nuclear magnetic resonance (NMR), are valuable tools for monitoring drug degradation (^1^H, ^13^C, ^19^F) [42]. These techniques can help identify functional groups and chemical bonds in the drug molecules, allowing researchers to track changes in the drug’s chemical structure during degradation. HPLC-MS and other hyphenated techniques (HPLC-NMR, UHPLC-Q-TOF/MS/MS, or APCI-MS) offer complementary information, combining the separation capabilities of HPLC with the structural elucidation power of MS or NMR and enabling a more comprehensive understanding of degradation mechanisms [10,77]. In a study by Wille et al. [96], 15 antidepressants, including AMT, were monitored, and indications regarding the indicated therapeutic dose (TDM) were provided. It was also acknowledged that side effects of AMT are best correlated with the concentration of its main metabolite, nortriptyline [96].

### 2.5. Stability-Enhancing Approaches

**Figure 2:**
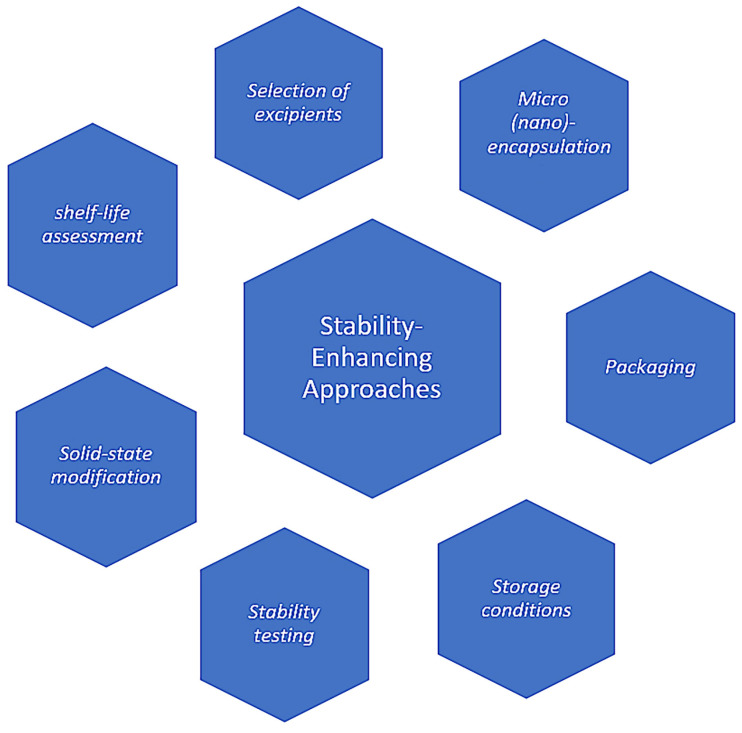
Main approaches used to enhance the stability of antidepressant drugs.

The stability-enhancing approaches for antidepressant drugs are aimed to mitigate chemical degradation and maintain drug quality, and they are essential in the development and formulation of antidepressant drugs to ensure their integrity, efficacy, and safety throughout their shelf life. The susceptibility of these drugs to degradation poses a significant challenge in maintaining their stability. To this end, various strategies and techniques have been employed to enhance the stability of antidepressant medications, including formulation optimization, packaging considerations, use of stabilizers, and advanced drug delivery systems (Figure 2). Formulation optimization plays a crucial role in enhancing the stability of antidepressant drugs. Factors such as drug–excipient compatibility, the selection of appropriate excipients and antioxidants, and the optimization of manufacturing processes are critical considerations. The use of excipients with stabilizing properties, such as antioxidants and moisture-barrier materials, can help protect the drug from degradation. Optimizing the formulation also involves selecting the most suitable dosage form and employing techniques such as matrix systems or controlled-release formulations to ensure a sustained drug release profile and minimize degradation [2,73].

To enhance the stability of antidepressant drugs, the selection of excipients and thoughtful packaging considerations play a key role in shielding antidepressants from external factors accelerating degradation. Incorporating desiccants and oxygen scavengers within the packaging creates a dry and oxygen-free environment, effectively reducing the risk of degradation. The impact of excipients cannot be overlooked, as incompatibility between drug substances and excipients can lead to degradation reactions. Formulation factors, including the presence of moisture, solvents, and processing techniques, also significantly influence drug stability [39,41].

Advanced drug delivery systems emerge as powerful tools for enhancing the stability of antidepressant drugs. These systems offer controlled drug release, improved solubility, and protection from degradation. Controlled-release formulations, such as extended-release tablets or transdermal patches, minimize drug exposure to degradative conditions, maintain therapeutic levels, and reduce dosing frequency. Nanoparticle-based drug delivery systems and lipid-based formulations contribute to enhanced drug solubility, bioavailability, and stability. These systems safeguard the drug from environmental factors and facilitate targeted delivery, thereby improving overall drug stability and efficacy [2].

Solid-state modifications involving changes in the crystalline structure or physical state of a drug substance significantly influence the stability, bioavailability, and efficacy of antidepressant drugs. Concerns arise regarding the transformation of the original crystalline structure into less stable or less bioavailable forms, such as polymorphs, solvates, hydrates, or amorphous forms. These modifications can alter dissolution rates, hygroscopicity, and chemical reactivity, impacting the drug’s absorption and bioavailability. Triggered by factors such as temperature, humidity, or light exposure during manufacturing, storage, or transportation, solid-state modifications can lead to drug degradation, diminishing the potency and therapeutic effectiveness. Understanding these mechanisms is imperative for designing robust and stable antidepressant formulations and ensuring safer and more effective treatments for mental health disorders [39,73].

The packaging and storage of antidepressant drugs hold paramount importance in ensuring their stability and efficacy throughout their shelf life. Effective packaging and storage conditions are crucial for preventing degradation, preserving drug quality, and safeguarding patient safety. Antidepressant drugs are vulnerable to various degradation mechanisms, such as hydrolysis, oxidation, and photodegradation [2,73]. The choice of appropriate packaging materials is critical for shielding the drug from moisture, light, and oxygen, factors that can accelerate degradation reactions. Packaging materials should form an effective barrier against moisture ingress while limiting the drug’s exposure to light and air. Stability testing and shelf-life determination involve simulating long-term stability by applying stress conditions (e.g., temperature, humidity, light/UV). This helps predict degradation pathways and identify degradation products, facilitating the determination of the drug’s shelf life [43].

These approaches ensure that antidepressant medications retain their therapeutic properties, minimize degradation-related risks, and offer reliable and effective treatment options. Employing robust stability-enhancing strategies is crucial for improving the stability of antidepressant drugs and optimizing patient outcomes. The significance of packaging and storage considerations is underscored by the need to select appropriate materials and design efficient containers, protecting antidepressant drugs from degradation throughout their journey from manufacturing to the end of their use [73].

### 2.6. Regulatory Considerations and Quality Control

The regulatory considerations and quality control measures are essential parameters associated with antidepressant drugs. The regulatory guidelines and requirements for drug stability assessment, the importance of complying with regulatory standards, and the implementation of robust quality control measures can ensure the safety, efficacy, and consistency of antidepressant medications [87,90]. Regulatory agencies, such as the U.S. Food and Drug Administration (FDA), the European Medicines Agency (EMA), and other regional regulatory bodies, establish guidelines and requirements that pharmaceutical companies must adhere to during the development, manufacturing, and distribution of antidepressant medications [71,73,77]. Regulatory agencies across different countries and regions strive to harmonize stability guidelines to facilitate global drug development and ensure consistent standards. The International Conference on Harmonization (ICH) guidelines, such as Q1A (R2) and Q1B, provide guidance on stability testing requirements, storage conditions, and shelf-life determination [89]. Harmonization promotes uniformity in stability testing protocols, facilitating data exchange among regulatory agencies [72,73].

Quality control is an integral part of ensuring the safety and efficacy of antidepressant drugs, and it involves quality control procedures throughout the manufacturing process, from raw material selection to the final product release [31,85]. Quality control measures include rigorous testing of raw materials, in-process testing during manufacturing, and finished product testing [56]. Analytical techniques such as chromatography, spectroscopy, and dissolution testing are employed to assess drug identity, purity, content uniformity, and dissolution characteristics. Compliance with quality control measures and adherence to Good Manufacturing Practices (GMPs) guarantees that the antidepressant drug product meets predefined quality standards [4,20,57,73]. Post-market surveillance is an integral part of regulatory considerations and quality control, and it involves monitoring the performance of antidepressant drugs after they enter the market [26]. Post-marketing stability studies, adverse event reporting, and pharmacovigilance programs help identify any stability issues, unexpected side effects, or safety concerns [25]. The QbD approach was successfully applied to AMT, allowing for more precise impurity profiling by forced degradation [97].

## 3. Degradation of Amitriptyline: Parameters, Products, and Kinetics

Several relevant case studies illustrate the degradation of antidepressant medications, focusing on one of the most prescribed tricyclic antidepressants (TCAs), namely, amitriptyline. By examining specific examples, one can gain insights into the degradation pathways, factors contributing to degradation, and potential strategies for mitigating degradation. Understanding drug degradation can guide the proper implementation of stability-enhancing approaches. Antidepressants (including TCAs) have been studied, and their decomposition products have been identified using various analytical techniques. Typical TCAs include amineptine, amitriptyline, amoxapine, clomipramine, desipramine, doxepin, imipramine, maprotiline, nortriptyline, opipramol, and trimipramine. Other antidepressants have been utilized in the therapeutic treatment of depression, such as fluoxetine and sertraline hydrochloride. Amineptine is metabolized through the beta-oxidation of the side chain, yielding a metabolite with 5 C atoms in the side chain, with relevant antidepressant pharmacological activity. Both clear from plasma within 10 h. Amitriptyline has a high thermal stability (up to 188 °C [11]); it can yield many degradation products, which will be discussed in more detail later. Amoxapine yields irreversible degradation in artificial gastric juice (following first-order kinetics, *Ea* = 88.70 kJ/mol; Δ*S_a_* = −80.73 J/K·mol) [93]. Clomipramine produces imipramine, HO-imipramine, desmethylclomipramine, and HO-imipramine-N-oxide through photodegradation, with a quantum yield of CMP degradation of 65.0 × 10^−3^ [98]. Desipramine is the major metabolite of imipramine and lofepramine. During the UV photodegradation of DES, a negligible influence of temperature on the decomposition rate was noted [84]. With high thermal stability (up to 211 °C [11]), DES can still yield some PTPs that could be mutagenic, genotoxic, or eco-toxic. Doxepin was detected in the water bodies from treatment plants and in surface water at a concentration in the range of 0.054–0.17 µg/L [2]. PTPs from doxepin show a phototoxic effect on erythrocytes [2]. Fluoxetine (FLU), a drug that was FDA-approved in 1987, produces two main photodegradation products, and both PTPs displayed some biological toxicity [99]. Other studies have also shown that the products of fluoxetine are present in food supplements [100]. Chemical oxidation of imipramine in an FeOCl-activated peroxymonosulfate process showed up to 64% degradation efficiency for imipramine [52]. Thermal degradation of IMI (imipramine) showed that it was stable up to 174 °C [11], but UV radiation induced demethylation and hydroxylation processes [101]. Maprotiline (MPT) can be regarded as either a TCA or tetracyclic antidepressant (TeCA), and it has been shown to be unstable under doubled ICH conditions (400 W m^−2^ and 2.4 × 10^6^ lx h) [2]. Liquid chromatography–high resolution mass spectrometry (LC/HRMS) (Acquity HPLC system, Water Technologies, Guyancourt, France, coupled with a Bruker SolarixXR FT-ICR 9.4 T MS instrument, Bruker Daltonics, Bremen, Germany) showed that MPT degradation using semiconductor photocatalysts (Fe-ZnO, Ce-ZnO, TiO_2_) was more efficient than heterogeneous photo-Fenton processes (H_2_O_2_, persulfate) [102]. Significant bioaccumulation (170 µg/kg) of MPT was observed in blue mussels with the formation of 12 intermediates resulting from hydroxylation/oxidation and ring opening [102]. Nortriptyline was detected as an effluent from WWTPs (wastewater treatment plants) in concentrations of about 13 ng/L [2] or 66 (effluents)–2092 ng/L (influents, UK) [6]. UV irradiation and high [H_2_O_2_] concentration increased its degradation rate. The recovery of NTRI (nortriptyline) by treatment plants in Canada was as high as 92 ± 1% [70]. The degradation of NTRI was fast in aqueous fulvic acid solution (k = 0.16 h^−1^) [53], but the acidity of artificial gastric juice did not cause any degradation of NTRI [94], which pointed out a different operating mechanism. A toxic effect on the aquatic system has been observed in the case of zebrafish (*Danio rerio*) by inhibiting the acetylcholinesterase activity, which provoked an anxiety-like state due to the impairment of the enzymes [20]. Retention of ~70% was achieved by using azide-functionalized silica-coated magnetite adsorbents—Fe_3_O_4_/SiO_2_/N_3_ [20]. Early-stage administration of 10 g activated charcoal could reduce NRTI absorption by 77% (after 30 min) [103]. Opipramol can be degraded through oxidation with a solution of manganese (III) pyrophosphate (P_2_O_7_^4−^), and five degradation products were detected in MS spectra [104,105]. Oxidative degradation in the presence of mild oxidants (salts of Mn(III)), as evidenced by the EPR spectra of desipramine–manganese(III) reaction mixtures in a 1:1 molar ratio, showed a reduction of Mn(III) to Mn(II), along with the respective oxidative degradation products [106]. Sertraline hydrochloride could be almost fully degraded (~99%) during prechlorination at WWTPs [75]. The effect on non-target organisms was evident through the ecotoxicological effects—more specifically, through sedimentary nitrification processes [4]—with significant bioaccumulation potential in wild fish (US) [7,56]. The degradation product desmethylsertraline has also been found in fish [56]. Trimipramine could be extracted with high efficiency (>81%) using the proper selection of a solid-phase extraction (SPE) method [69]. The teratogenic effects of trimipramine (TMP) and its derivatives have also been assessed; 14 such PTPs were reported in the same study [85]. Electrospray ionization quadrupole ion-trap mass spectrometry (ESI–MS) was used to characterize TMP degradation products, and TMP was also used as an internal standard for the quantification of degradation products of other TCAs [13,26,107]. Antidepressant drugs such as sertraline hydrochloride [2,77] or fluoxetine [99] act as selective serotonin reuptake inhibitors (SSRIs), and their degradation pathways are dominated by forced hydrolysis and UV exposure.

A brief summary of the most common TCAs, alongside their chemical formulas, degradation products, and mechanisms of action, is given in Table 1.

While the family of TCA drugs offer a fascinating perspective on drug degradation routes, the current review aims to discuss the chemical degradation of amitriptyline in part due to the sheer number of reports dealing with AMT degradation and due to the rich chemistry displayed by AMT during degradation studies, including AOPs (advanced oxidation processes) (Table 2).

**Figure 3:**
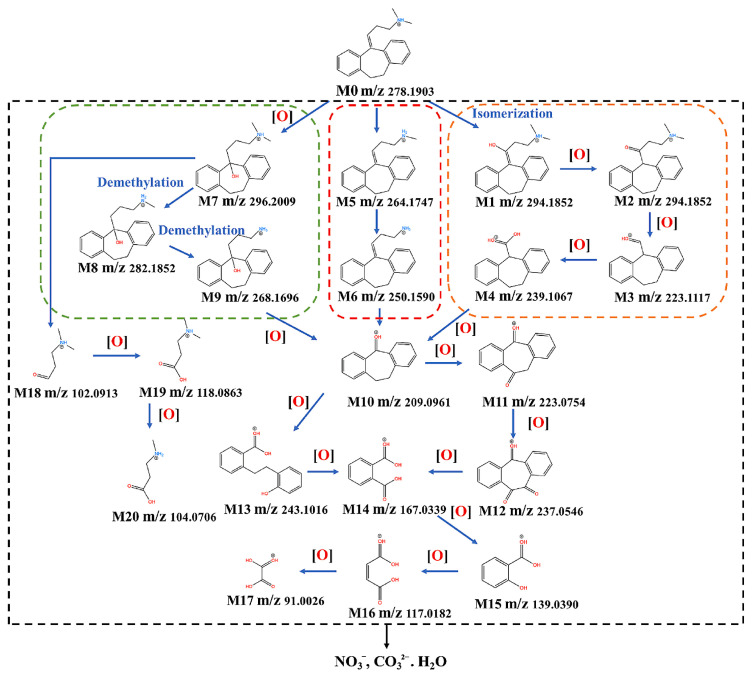
Degradation pathway of amitriptyline (AMTH) using a CoFe-LDH catalyst. Reproduced from reference [47].

Amitriptyline’s degradation in its protonated form (AMTH) was investigated using ESI-QTOF MS (electrospray ionization quadrupole time-of-flight mass spectrometry) in a heterogeneous electro-Fenton system based on a layered double hydroxide structure (LDH) (Figure 3) [47]. The transformation of AMTH (M0, *m*/*z* 278) into M1 (*m*/*z* 294) is the result of the oxidative hydroxylation of the olefin bond. The isomerism M1/M2 is an enol–keto tautomerism that, under further oxidation, leads to the removal of the branched chain, the formation of aldehyde M3 (*m*/*z* 223), and, even further, the acid M4 (*m*/*z* 239). Decarboxylation of M4 yields ketone M10 (*m*/*z* 209). Another reaction path leads to products M5 and M6 through the demethylation of M0, which could undergo attack from hydroxyl radicals to M10. Lastly, a third pathway implied hydroxylation of amitriptyline to M7, and further oxidation with consecutive demethylation yielded M8 and M9; further oxidation led to ketone M10. This ketone M10 could undergo a ring-opening reaction to produce M13 and, upon further oxidation, ortho-phthalic acid M14 and ortho-hydroxybenzoic acid M15. Further oxidative degradation of the above products would lead to M16 maleic acid and M17 oxalic acid by cracking the benzene ring of M15. The branched chain of M7 could also be removed to obtain M18 and be further oxidized to obtain M19 and M21.

**Figure 4:**
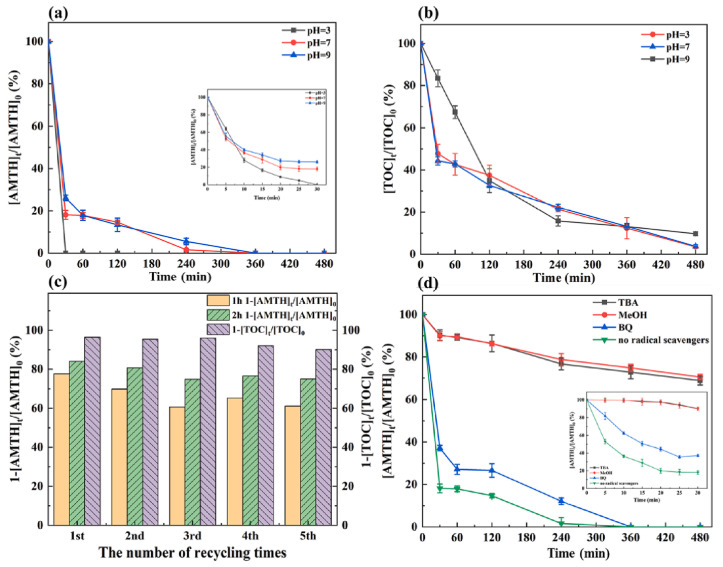
(**a**) Decontamination of AMTH at pH 3, pH 7, and pH 9 with an LDH/CF cathode. (**b**) TOC mineralization efficiency at different pH levels. (**c**) The long-term (5 runs) stability of the CoFe-LDH/CF cathode. (**d**) Quenching experiments (the inset tracks the decomposition in 30 min). Reproduced with permission from Ref. [47].

Notably, all mentioned reactions lead to complete mineralization into small molecules such as H_2_O, CO_2_, NH_4_^+^, and NO_3_^−^, which are feasible due to the fact that AMTH has a nitrogen atom in its structure. In fact, the total amounts of quantified nitrogen correspond to 99.44% (6.165 mg/L) of the initial nitrogen of 0.1 mM AMTH, which indicates the highly effective oxidation reaction pathways [47]. Using a CoFe-LDH/CF cathode for AMTH removal and mineralization seems to be highly efficient, as the catalyst showed similar activity even after five runs (Figure 4) [47]. The CoFe-LDH/CF cathode showed a minor decay in performance over five runs (Figure 4c), while the degradation of AMT tracked over 480 min showed a faster degradation route under more acidic pH values. Typical quenching experiments are aimed at elucidating the mechanistic insights of degradation (Figure 4d). Specific quenching reagents can be used in order to better quantify which of the proposed mechanistic proposals is feasible; in the case of CoFe–DLH, three scavengers were used: tertiary butanol (^t^BuOH, traps •OH), methanol (CH_3_OH, traps •SO_4_^−^ and •OH), and benzoquinone (BQ, •O_2_^−^ removal). The nature of ROSs (reactive oxygen species) could be better attested by these scavenging experiments: BQ basely affected the reaction rate, whereas ^t^BuOH and CH_3_OH addition significantly decreased the rate, indicating that hydroxyl radicals •OH are more likely to be the main reactive species in the LDH-based hetero-EF system [47].

A study conducted by M. Wu et al. on antidepressants found in Huangpu River (Shanghai, China) showed that the recovery rate of AMT was high in a wastewater treatment plant (WWTP) at 90 ± 4% [69]. It was only fluoxetine, venlafaxine, and amitriptyline that were found above the LOD in the mentioned study [69]. On the other hand, there are indications that microorganisms from the secondary treatment of wastewater in WWTPs could be responsible for an increase in antidepressant concentration, as they return to the environment in their initial form [4]. Other studies on biosolids from Canada used and validated LC-MS/MS analytical methods to identify a maximum concentration of AMT of 768 ng/g in selected sewage samples that were collected [70].

**Figure 5:**
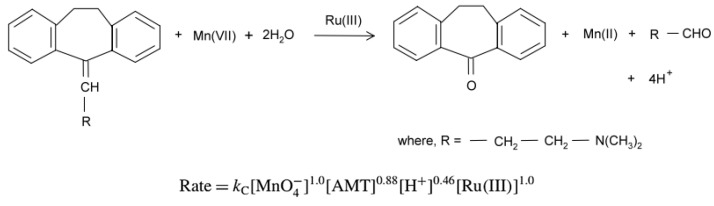
Reaction of AMT with KMnO_4_/H+ media in acidic media under [Ru(H_2_O)_6_]^3+^catalysis [108].

Chemical oxidation of AMT using a Ru(III)./KMnO_4_/H^+^ medium led to a 1:1 molar consumption of AMT:MnO_4_^−^ [108]. Employing a Ru(III) catalyst (active form: [Ru(H_2_O)_6_]^3+^) showed an eight-fold reaction rate increase compared to the uncatalyzed reaction, and the final identified products were *trans*-4-aminocyclohexanal and dibenzosuberone. Kinetic measurements revealed the partial reaction order with respect to [MnO_4_^−^] and [Ru(III)] to be 1; meanwhile, the fractional reaction orders were deduced for AMT (n = 0.88) and [H^+^] (n = 0.46) (Figure 5).

**Figure 6:**
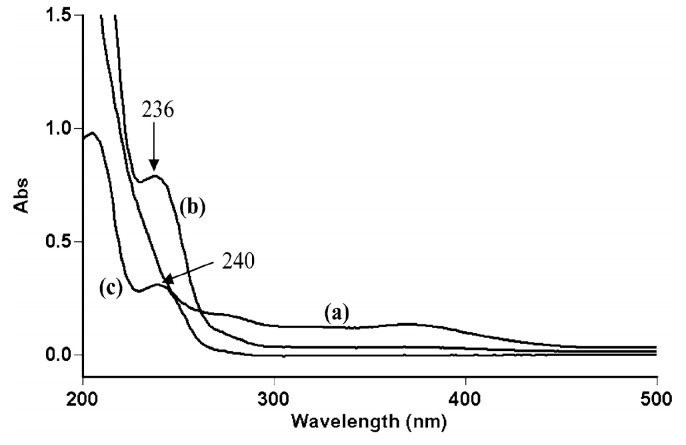
Spectroscopic evidence (UV-Vis) of complex formation between Ru(III) and AMT for (*a*) Ru(III), (*b*) a mixture of Ru(III) and AMT (236 nm), and (*c*) AMT (240 nm). Reproduced with permission from Ref. [108].

The reaction mechanism implies the formation of a Ru(III)-organic substrate complex, which has been evidenced by spectroscopic measurements, where a clear difference between UV-Vis spectra was observed for the AMT–Ru(III) mixture, consistently with the formation of the aforementioned complex (Figure 6) [108].

**Figure 7:**
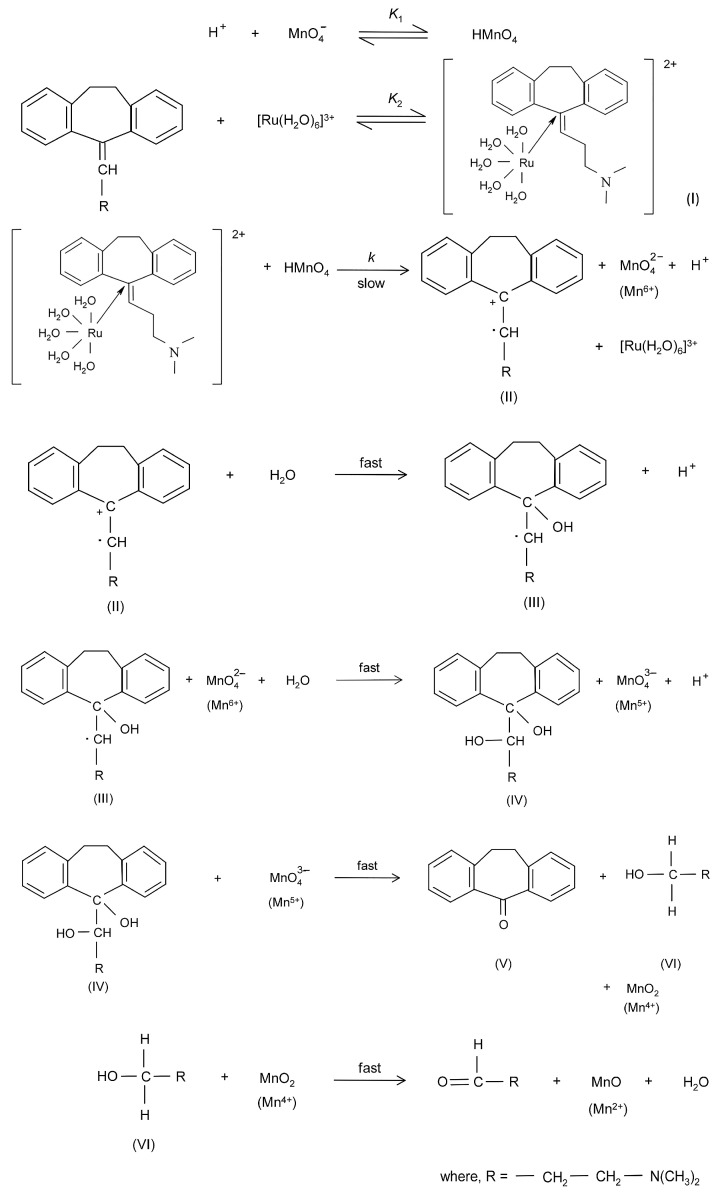
Detailed mechanism of AMT oxidation by permanganate ions with the Ru(III) catalyst [108].

A detailed mechanism for the degradation of AMT in the presence of Ru(III) catalyst was proposed by the authors (Figure 7) [108]. The proposed mechanism depicted in Figure 7 comprises the formation of HMnO_4_ as active an oxidant species (K1), a step where H^+^ and MnO_4_^−^ react to form permanganic acid. A second step involves the formation of the complex Ru(III)–AMT, which further reacts with HMnO_4_ to form the carbocation of AMT radical (II), MnO_4_^2−^ (Mn^6+^), and H^+^ species with the regeneration of the catalyst, the octahedral–coordinated ruthenium(III) species, [Ru(H_2_O)_6_]^3+^. In a fast step, the carbocation of the AMT radical (II) hydrolyzes to give the intermediate radical (III) and H^+^. In a further fast step, the intermediate compound (III) reacts with MnO_4_^2−^ to give the vicinal diol (IV), generating MnO_4_^3−^ (Mn^5+^) and H^+^ species. The diol (IV) reacts with MnO_4_^3−^ to give dibenzosuberone (V), 2-amino-3-methyl-1-butanol (VI) and MnO_2_ (Mn^4+^). In the last step, 2-amino-3-methyl-1-butanol (VI) reacts with the MnO_2_, which acts as a mild oxidant and is, therefore, oxidized to the aldehyde species *trans*-4-aminocyclohexanal (VII). Consequently, MnO_2_ (Mn^4+^) is reduced to MnO (Mn^2+^) [108].

As an emerging contaminant, AMT has been tested on a number of in vivo models, such as marine and freshwater snails (increased righting time in snails), male albino rats (liver and kidney damage), fish and frog embryo, fish larvae (causing effects such as swimming alterations, affecting mRNA expression of genes related to the heart, eye, brain, and bone, increasing lipid peroxidation, and causing morphological anomalies or pathological changes in key organs such as the brain, heart, or kidney) [6]. These studies outline the dangers of AMT as a representative example of TCA in aquatic ecosystems that can be easily disturbed by pollution. Current removal techniques for AMT include photocatalytic systems using Co-doped titanate nanowires, Mn(III)-permanganate oxidation systems, granular active carbon, or photocatalytic sol-gel systems, affording a removal rate of 41 to >97% [6,108]. Photocatalytic systems usually afford the highest removal rate (>90%). Some authors suggest that the presence of AMT and TCAs in general in remote areas is related to the presence of tourists at the respective sites [6].

**Figure 8:**
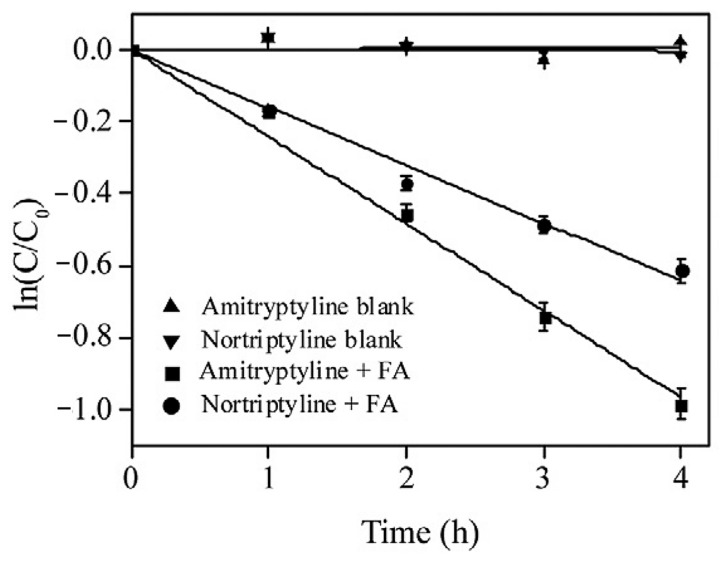
Photodegradation kinetics of amitriptyline and nortriptyline with or without fulvic acid. Reprinted with permission from Ref. [53].

While resistant to photodegradation, AMT (k = 0.24 h^−1^) and its metabolite nortriptyline (k = 0.16 h^−1^) underwent decomposition following pseudo-first-order kinetics when decomposed in air-saturated fluvic acid (FA) solution under basic conditions (pH = 8) [53]. Starting from a drug concentration of 10.0 mM, the kinetics were followed over 4 h in the absence and presence of fulvic acid (FA, 20 mg L^−1^). In the absence of FA, there was no reactivity of either amitriptyline or nortriptyline (Figure 8) [53].

**Figure 9:**
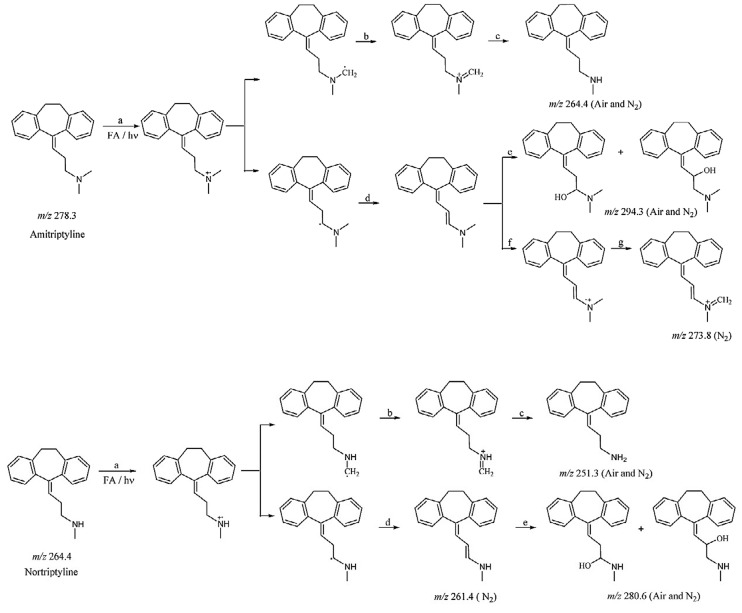
Photodegradation routes of AMT and nortriptyline in fulvic acid (FA) solution. Reproduced from Ref. [53].

Since acidity alone led to no degradation products, the AMT underwent a different degradation mechanism in which the FA* (activated species) played a key role (Figure 9). The electron transfer mechanism proposed by the authors of [53] was based on quenching experiments, the influence of pH, and the kinetic model that they developed. The key step seems to be the electron transfer from the non-bonding N-electrons of AMT/nortriptyline to the excited FA triplet states (Figure 9).

The process is initiated by reaction (a) comprising the said electron transfer, followed by the fast transfer of α-H to yield a radical carbon intermediate ((b), (d)). The process (b) leads to the formation of an unstable C=N radical state, which, upon cleavage, produces demethylation to the final product, nortriptyline (process (c), *m*/*z* 264.4 for AMT). Process (d) leads to the dehydrogenation of the radical carbon intermediate and the formation of intermediates featuring conjugated double-bond systems. Demethylation and hydroxylation are the two main degradation processes for the photosensitized degradation of amitriptyline and nortriptyline in the FA solution [53].

**Figure 10:**
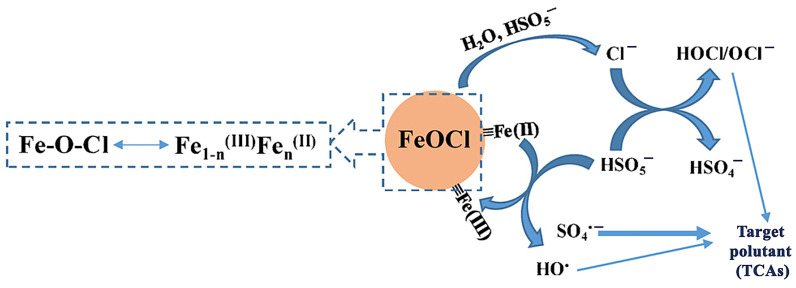
Degradation mechanism for the FeOCl/PMS catalytic system. Modified from [52].

Other catalytic systems such as iron oxychloride (FeOCl)/peroxymonosulfate (PMS) have been employed in the degradation of target pollutants and other organic compounds such as (alkyl)parabens, amitriptyline, desipramine, or propranolol (Figure 10) [52].

**Figure 11:**
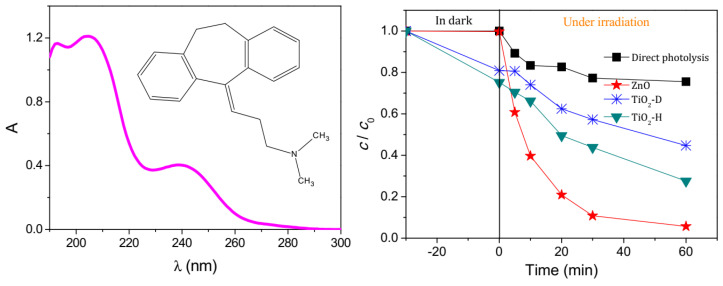
Absorption spectrum of AMI (amitriptyline hydrochloride, 0.03 mM) (**left**) and the kinetics of AMI photolysis and photocatalytic degradation (**right**). Reproduced from Ref. [50] under a Creative Commons Attribution (CC BY) license.

Given the high reactivity of the radical anion SO_4_**^·−^** towards organic compounds, the catalytic system FeOCl/PMS seems to be a promising route affording their high-yield degradation. Another commonly employed photocatalytic system based on ZnO (41 nm, 0.016 cm^3^/g, 6.5 m^2^/g) has been utilized for the removal of organic contaminants, including TCAs such as AMT. Under optimized parameters (1 mg ZnO/mL, [AMT] = 0.03 mmol/L, at 25 °C, pH = 6.7, 60 min) and solar irradiation, ZnO afforded 94.3% removal of amitriptyline (Figure 11) [50,91]. Other well-established photocatalysts such as TiO_2_ (anatase/rutile) have also been screened in this reaction: TiO_2_-D (75% anatase, 25% rutile, 20 nm, 0.134 cm^3^/g, 53.2 m^2^/g) and TiO_2_-H (anatase, 35–65 m^2^/g) [50].

**Figure 12:**
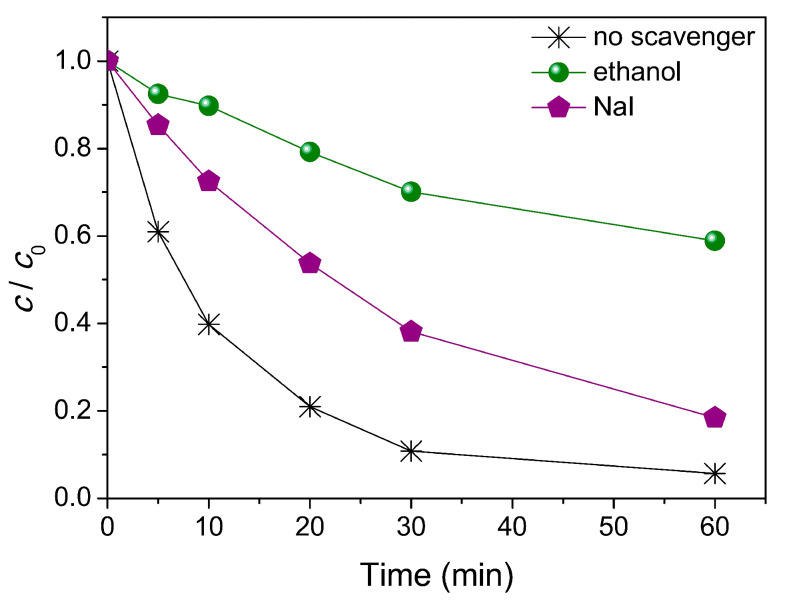
The effects of different quenching reagents (NaI or C_2_H_5_OH) on the kinetics of AMI photodegradation. Reproduced from Ref. [50] under a Creative Commons Attribution (CC BY) license.

The results reported by Fincur et al. [50] show that ZnO had better activity when compared to TiO_2_ catalysts, which can be attributed to the better mobility, generation, and separation of e^−^-h^+^ pairs from ZnO. There was no degradation of AMI after 850 days in the dark, so the presence of a photocatalyst was compulsory [50]. Figure 11 also shows that there is an absorption process occurring in the dark, where ZnO adsorbs no AMI in the dark, whereas TiO_2_-D adsorbs 19.1% of AMI and 24.8% of TiO_2_-H within 30 min [50]. The addition of electron acceptors (O_2_, KBrO_3_, H_2_O_2_, (NH_4_)_2_S_2_O_8_) typically leads to improved activity over time for photocatalysts, since this inhibits the recombination of e^−^-h^+^ pairs, increasing the radical concentration [HO·] (Figure 12).

In the presence of scavengers such as ethanol (scavenger of •OH radicals) and NaI (with I^–^ being a scavenger for adsorbed •OH radicals and photogenerated holes), the reaction slowed down considerably, and even more so when C_2_H_5_OH was added, which hinted at quenched reactivity of free hydroxyl radicals, which are, therefore, thought to be the main photocatalytic degradation pathway, and reactive holes are probably a secondary mechanism [50]. Ultimately, the mineralization of AMI occurred to a degree of about 30% after 180 min of irradiation, producing inorganic species such as NH_4_^+^, NO_3_^−^, NO_2_^−^, CH_3_COO^−^, and HCOO^−^.

**Figure 13:**
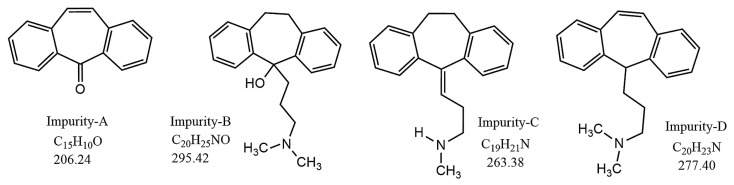
The four main impurities typically found in AMT hydrochloride. Reproduced with permission from [87].

As the active component of various drugs used to treat anxiety symptoms, as well as depression and schizophrenia with depression, AMT has been tested with respect to stability under various conditions of forced degradation [5 M HCl at 80 °C/1 h, 5 M NaOH at 80 °C/1 h, H_2_O (*v*/*w*) at 80 °C/1 h, 6% H_2_O_2_ (*v*/*v*) at 25 °C/1 h, dry heat at 105 °C/24 h and UV–vis light/4 days] [87]. Boppy et al. analyzed and quantified the four main impurities found in amitriptyline hydrochloride tablets using HPLC by correlating known degradation pathways of AMT with mass spectral data (*m*/*z*) (Figure 13) [87].

**Figure 14:**
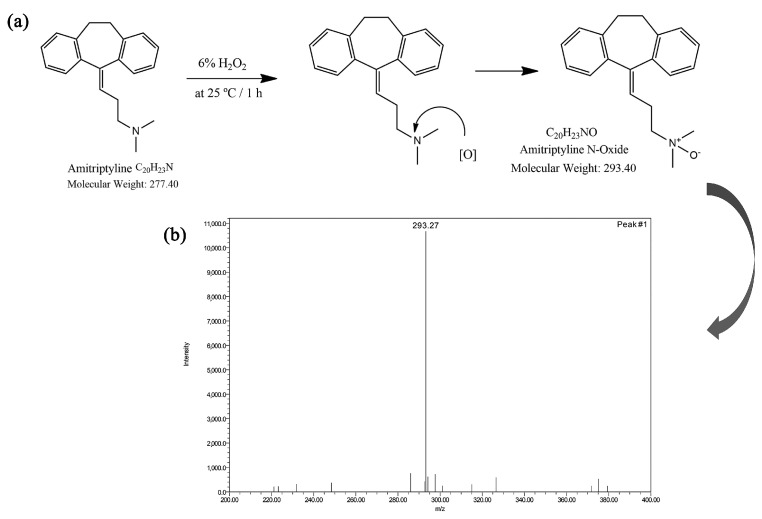
(**a**) Degradation mechanism in the oxidation of AMT with H_2_O_2_ and the representative LC–MS spectra of a newly formed degradation product, amitriptyline analog (N-oxide); (**b**) LC-MS spectra for degradation product. Reproduced with permission from [87].

Using methanol in the mobile phase and a validated method coupling HPLC with MS, the authors investigated the stress degradation process of AMT regarding hydrolysis stress, acid hydrolysis stress, base hydrolysis stress, oxidative stress, and photolytic stress, as well as thermal stress testing under open exposure and dry heat conditions (Figure 14) [87].

When H_2_O_2_ (6%, oxidant) was added to a solution of 100 mg AMT and the reaction continued for 1 h, the mechanism proposed was based on active oxygen [O] attack at the electron-rich N-center of AMT, yielding the corresponding AMT N-oxide (Figure 14). The LC-MS spectrum of the fraction collected from the oxidation of AMT (in negative scanning mode) at RT 6.2 min confirmed the formation of amitriptyline analogue N-oxide (*m*/*z* 293.27). Moreover, the method was validated under ICH Q2(R2) guidelines and USP <1225>, affording a minimum resolution of 1.5 without drifting or interference, and all four degradation products were separately eluted (Figure 13). The method presented could be used in the lab testing and stability assessment of AMT tablets [87].

Ledeti et al. studied the solid-state decomposition of AMI using the ICTAC 2000 recommendations while utilizing four methods (Flynn–Wall–Ozawa, Kissinger–Akahira–Sunose, Friedman, and the nonparametric kinetic method (NPK)), which indicated the apparent activation energy for decomposition in case of AMI to be Ea = 82.9 kJ/mol, the lowest among the three TCAs investigated by the authors (Ea = 123.4 kJ/mol for imipramine and Ea = 112.3 kJ/mol for desipramine) [11]. ATR-FTIR data showed the expected band positions for AMI: 3100–3000 cm^−1^ (aromatic CH stretch), 3000–2800 cm^−1^ (asymmetric and symmetric CH stretching from CH_2_/CH_3_ groups), 2600–2400 cm^−1^ (protonated amines as hydrochloride salts), and 800–700 cm^−1^ (benzene ring substitution) [11]. Overall, the presence of C=C bonds in AMT makes it less stable and more susceptible to degradation, especially via oxidation processes.

**Figure 15:**
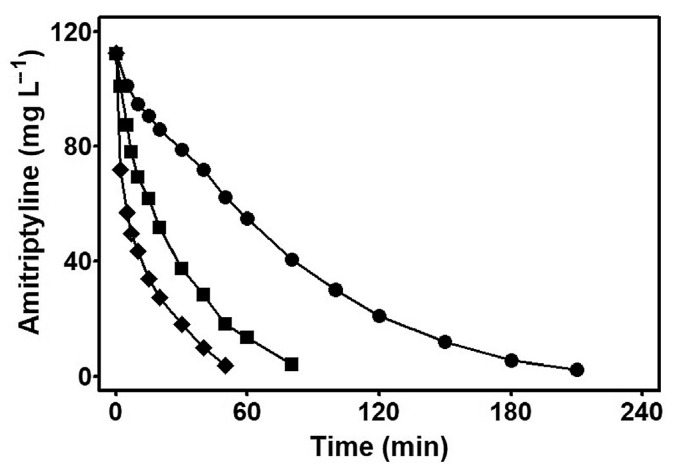
Amitriptyline hydrochloride concentration decay in a stirred tank reactor with BDD/ADE. AO-H_2_O_2_ (●), EF with 0.5 mmol/L (■), and PEF with 0.5 mmol/L (◆). Reprinted with permission from Ref. [51].

On the other hand, there are reports about AMT hydrochloride showing antibacterial properties in in vitro and in vivo experiments in mice [76]. AMT and its metabolite also reduced IL-1β and TNF-α secretion in LPS-activated mixed glia and microglia cultures [66]. Amitriptyline can be removed by bioreactors with biomembranes in a fast and effective process with a removal yield of greater than 80% [49,76]. TCAs and other organic products that can exist in their protonated form are easier to degrade, and this is also valid for AMT. However, amitriptyline (AMT) is one of the most commonly used TCAs available on the market, and it is considered a recalcitrant emergent pollutant due to its persistence on various water bodies, where it has been detected in several accounts (Figure 15) [51]. When treating AMT in a stirred tank reactor (130 mg/L) with 0.050 mol/L Na_2_SO_4_ at 100 mA/cm^2^, pH 3.0, and 35 °C, a faster decay in its concentration was observed with PEF with 0.5 mmol/L than with EF with 0.5 mmol/L or BDD/ADE. AO-H_2_O_2_.

**Figure 16:**
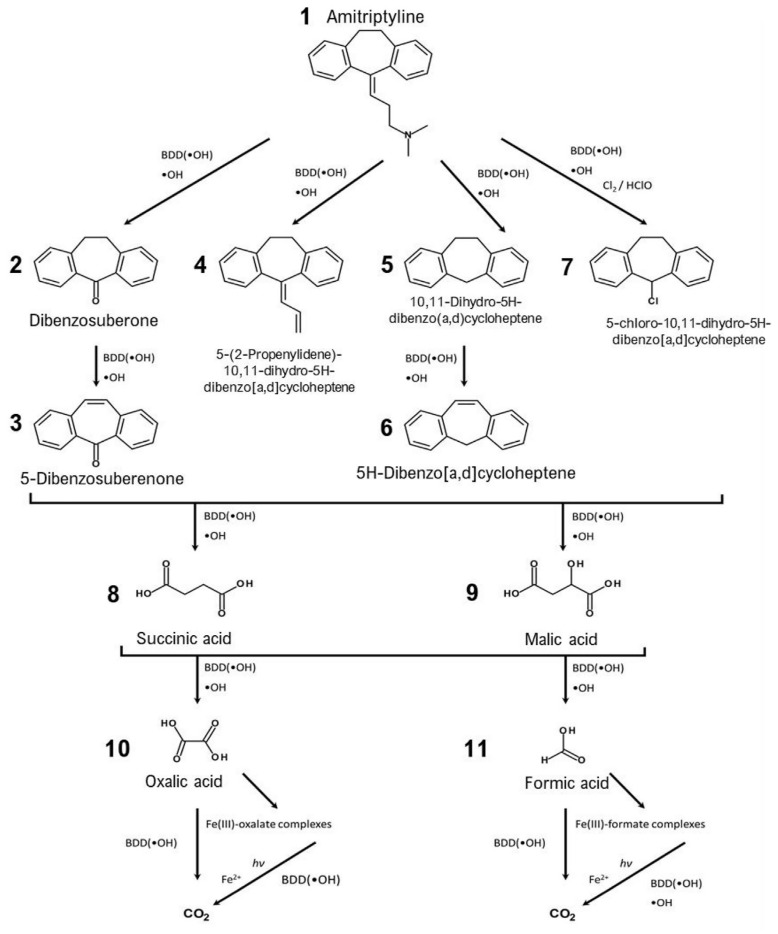
Proposed reaction sequence for the mineralization of amitriptyline by EAOPs using a BDD anode. Reprinted with permission from Ref. [51].

Advanced oxidation processes (AOPs) usually involve the use of hydroxyl radicals (·OH) and include electrochemical methods using the anodic oxidation of H_2_O (AO-H_2_O_2_), electro-Fenton (EF) reactions, or photoelectro-Fenton (PEF) reactions (Figure 16) [51]. Following a first attack on the olefinic chain of AMT (1), four compounds are formed, with three losing the alkyl group completely (dibenzosuberone 2, 10,11-dihydro-5H-dibenzo[a,d]cycloheptene 5 and 5-chloro-10,11-dihydro-5H-dibenzo[a,d]cycloheptene 7), while the fourth one undergoes oxidation at the carbon near the -N(CH_3_)_2_ functional group to afford a conjugated diene system: 5-(2-propenylidene)-10,11-dihydro-5H-dibenzo[a,d]cycloheptene (4). In a second oxidation stage, only two products form, with both containing an olefinic bond in the former cycloheptene backbone: 5-dibenzosuberenone (3) and 5H-dibenzo[a,d]cycloheptene (6). A third step of oxidation by hydroxyl radicals reduces the complexity of the products to only C4-compounds, succinic acid (8) and malic acid (9), which further undergo oxidation to oxalic acid (10) and formic acid (11), respectively. These simple carboxylic acids are then oxidized by either ·OH or photochemically degraded to CO_2_ by means of Fe(III)-oxalate or Fe(III)-formate catalysts (Figure 16).

**Figure 17:**
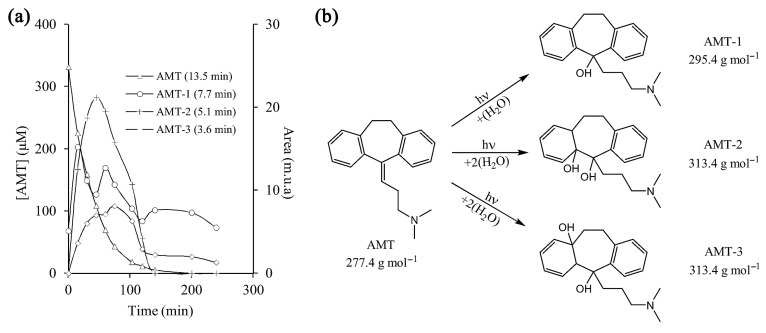
(**a**) Evolution of AMT concentrations and photoproduct areas as a function of irradiation (254 nm) time. (**b**) Structures of AMT and its products generated under UV irradiation in purified water. Reprinted with permission from Ref. [93].

Another insight into the photodegradation of AMT comes from Nassar et al., who studied the degradation of two antibacterial sulfonamides, namely, sulfamethazine (SMT) and sulfamethoxypyridazine (SMP), and two tricyclic antidepressants—amitriptyline (AMT) and clomipramine (CMP)—using artificial sunlight conditions while noting no acceleration in case of AMT (in contrast with the acceleration of the reaction rate by up to eight times for the other three investigated compounds) when conducting experiments in river water as compared to purified water (Figure 17) [93]. The calculated quantum yield in the case of AMT was 7.6 × 10^−3^ when AOP was used (UV/H_2_O_2_), while the second-order rate constant in the reaction of the organic substrate with •OH radicals was 8 × 10^9^ L mol^−1^ s^−1^ [93]. The products arising from these AOPs were assessed and identified by means of LC–MS/MS analyses.

**Figure 18:**
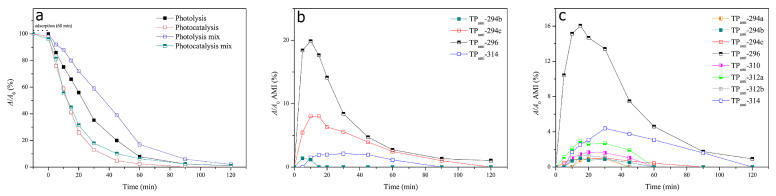
(**a**) Photodegradation of AMI in a single and mixed solution; (**b**) time profiles of AMI TPs during photolysis; (**c**) time profiles of AMI TPs in photocatalysis. Reproduced with permission from Ref. [16].

UV-Vis radiation produced the photocatalytic degradation of amitriptyline by utilizing cobalt–titanate nanowire-based nanocatalysts (Co-TNW) [16]. Respective degradation products (transformation products, TPs) were identified by using ultra-high performance liquid chromatography coupled with quadrupole time-of-flight mass spectrometry (Figure 18).

**Figure 19:**
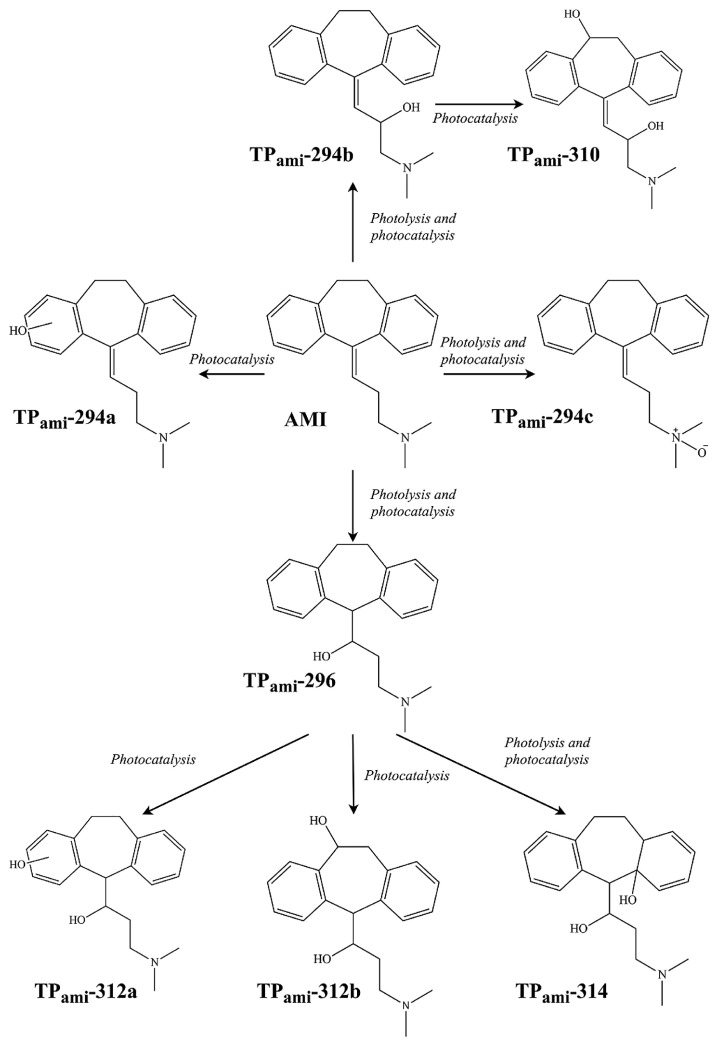
Degradation pathways proposed for AMI photolysis and photocatalysis. Reproduced with permission from Ref. [16].

Figure 18b shows the degradation of AMI under photolysis, whereas the degradation time profile of AMI during photolysis is shown in Figure 18c. Eight TPs have been elucidated in the case of AMI, and the effect of Co-TNWs was a clear enhancement in efficiency of AMI removal. Some of the TPs had even higher toxicity than that of the parent compound, highlighting the need to consider the toxicity of TPs to the ecosystem, as has already been confirmed by in silico studies [16]. The mechanistic insight presented in Figure 19 describes the degradation pathways leading to the eight confirmed TPs. It seems that this oxidation process is less energic than using Fe(III)-based complexes [51], producing degradation mainly in the AMT backbone and aliphatic chain without completely removing the propylidene moiety. With the exception of the N-oxide derivative of AMT, most identified TPs are generated through hydroxylation processes with varying degrees of substitution (one or two hydroxyl groups) both in the aromatic rings and in the backbone or aliphatic chain. However, the proposed structures even include an intermediate TP_AMI_-314 that loses aromaticity in one of the former phenyl rings due to hydroxylation [16]. Either way, this oxidation pathway is rather mild and will not cause the mineralization of AMT.

**Figure 20:**
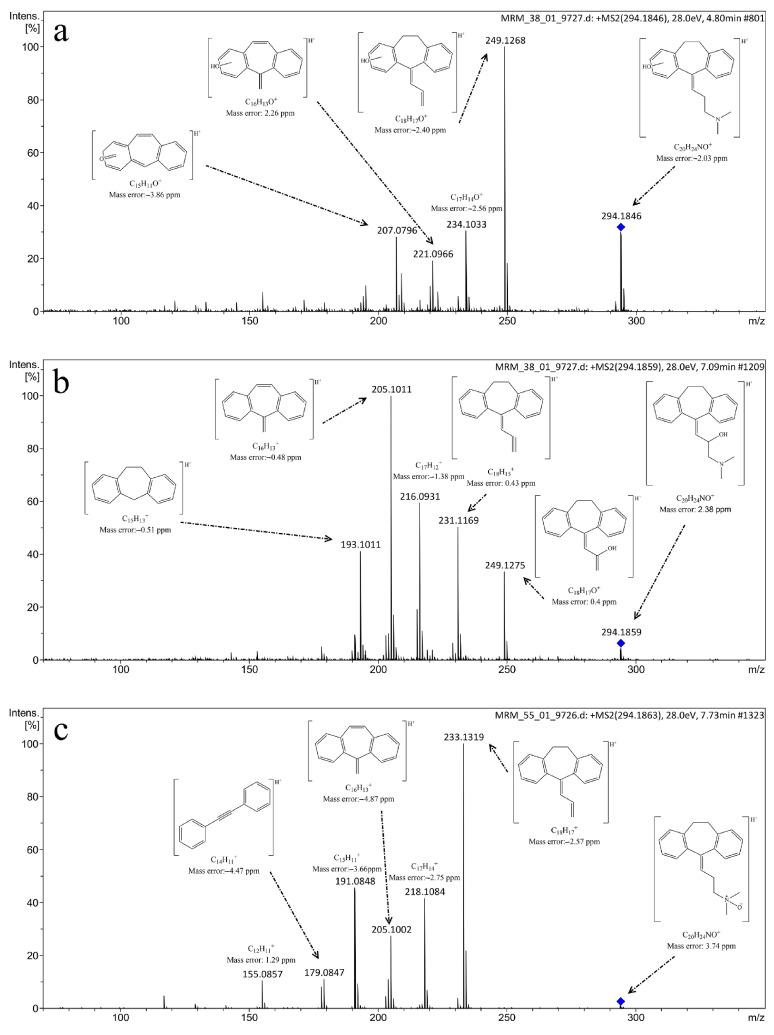
MS/MS spectra and proposed fragmentation of (**a**) TPami-294a, (**b**) TPami-294b, and (**c**) TPami-294c. Reproduced with permission from Ref. [16].

The photocatalytic degradation of AMI produced four TPs, but only four of them were present during photolysis, which implies that more complex oxidation mechanisms are at play during photocatalytic processes [16]. TP_ami_-294c, for instance, was degraded faster in the presence of Co-TNWs, thus proving the catalytic activity of Co-TNW for some AMI TPs as well. Not all TPs were degraded within the a 120-minute timespan, with TP_ami_-296 still being present in the reactive mixture, producing three new TPs, namely, TP_ami_-312a, TP_ami_-312b, and TP_ami_-314, upon further exposure. More hydroxylation reactions occurred during the photocatalysis, since the only identified TPs were TP_ami_-310, TP_ami_-294a, TP_ami_-312a, and TP_ami_-312b (Figure 19 and Figure 20). Corroborating the MS spectra with the proposed degradation pathways, two main processes appeared to occur during photodegradation, namely, hydration of the alkene moiety in the AMI structure and multiple hydroxylation processes (Figure 20). The MS spectrum analysis indicates the formation of a degradation product (TP_ami_-296) through the hydration of alkene in amitriptyline (AMI), along with the elimination of H_2_O, as indicated by the product at *m*/*z* 278 (C_20_H_24_N^+^). The proposed water elimination occurred on the γ-carbon of the tertiary amine, which was consistent with previous findings. There was no elimination of the C_2_H_7_NO fragment; therefore, the formation of N-oxide byproducts was precluded in this case.

Additionally, two subsequent degradation products (TP_ami_-312a and TP_ami_-312b) were likely formed by the hydroxylation of TP_ami_-296, which involved the hydration of alkene and hydroxylation processes. The MS spectra suggest the presence of hydroxyl groups on both the aromatic ring and the unsaturation of the aliphatic chain. The analysis, while not identifying the exact position of the OH group, implies a complex degradation pathway involving multiple hydroxylation events [16].

**Figure 21:**
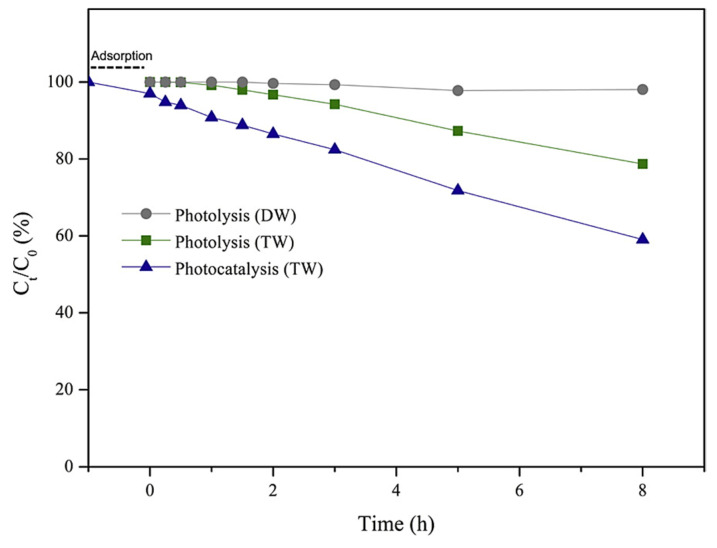
Photolysis and photocatalytic degradation of amitriptyline (AMI) in an aqueous solution. Reprinted with permission from Ref. [109].

Osawa et al. expanded the original study with further kinetic data in an upcoming research article [109]. The pseudo-first-order kinetics for photocatalysis (*k_ap_* = 0.0617 h^−1^) and photolysis (*k_ap_* = 0.0366 h^−1^) have been deduced and experimentally confirmed. Using the same Co-TNW catalyst, the degradation is effectively twice as fast. Conducting the degradation reactions in pure water (DW, distilled water) seems to be the least favorable choice, as there is basically no reaction in this reaction medium (Figure 21).

**Figure 22:**
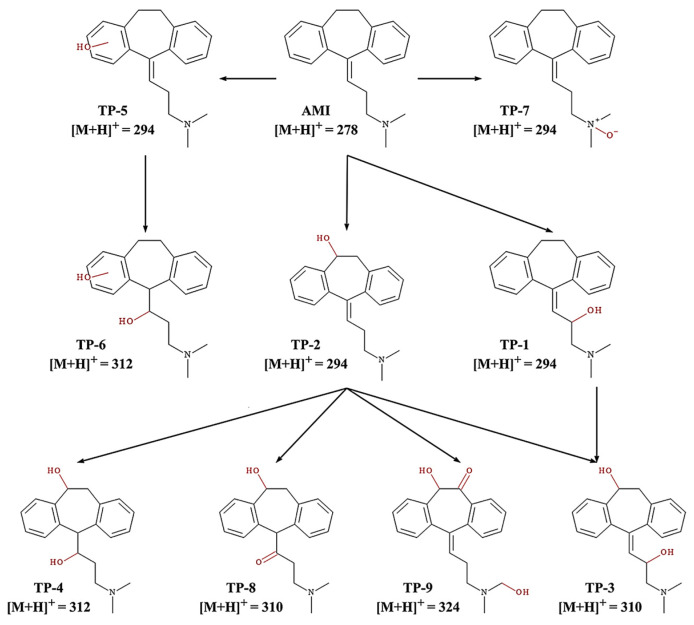
Proposed degradation pathways of amitriptyline (AMI). Reprinted with permission from Ref. [109].

TP-1 was generated through the hydroxylation of the β-carbon of the aliphatic chain, and TP-2 had the OH group attached to cyclohexane because neutral loss of H_2_O was detected (*m*/*z* 276, C_20_H_22_N^+^) (Figure 22) [109].

TP-3 could be produced through the hydroxylation of TP-1 or TP-2 (two water elimination events from the MS spectrum). In TP-4, the hydration of the aliphatic chain of TP-2 occurred. TP-5 was the result of a hydroxylation process, with an uncertain position of the hydroxyl moiety on the phenyl ring, and the hydration of TP-5 aliphatic alkene produced TP-6. In this investigation, the N-oxide product of AMI was detected (TP-7). TP-8 could originate from TP-2, which underwent oxidation on the alkene group, with the hydroxyl group potentially being bound to the cycloheptane ring. Lastly, TP-9 showed the elimination of CO in the MS spectrum, as well as the loss of C_2_H_7_NO and H_2_O, which supported two hydroxylation events—one at the tertiary amine carbon and one at the cycloheptane [109].

Saito et al. investigated the decomposition of AMT under forced hydrolysis conditions using artificial gastric juice (2 g NaCl, 7 mL HCl, and the rest up to 1000 mL purified water, pH = 1.2) as a reaction medium to better simulate the integrity of AMT once ingested orally [94]. Upon storage at the biologically relevant temperature of 38 °C for 25 days, almost no degradation occurred in the case of AMT. Amoxapine (AMX) was the only one of the eight compounds investigated that actually underwent decomposition in the artificial gastric juice [94].

HPLC was used in a reversed-phase method with ion pairing with pentanesulfonic acid in a C-18 column, allowing the simultaneous analysis of a combined mixture of TCAs, including AMT [112]. The importance of knowing the exact concentrations of antidepressants and those of their main decomposition products is vital for trained psychiatrists to offer the most effective treatment plans, as well as to better interpret patient outcomes while receiving relevant feedback. In this regard, a comprehensive profile of antidepressants in serum is essential. Patients receive not only antidepressants but also sedatives and/or tranquilizers; hence, all the possible interferences generated by these drugs must be accounted for in therapeutic treatments, which need to be evaluated as a whole to better correlate patients’ clinical responses [112].

**Figure 23:**
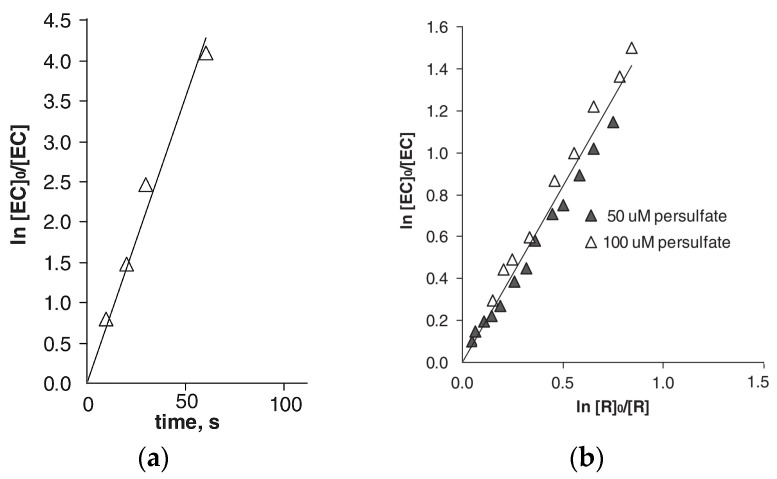
(**a**) Oxidation of AMT using the UV/S_2_O_8_^2−^ system in deionized water. (**b**) Determination of the second-order rate constants. Reproduced with permission from Ref. [36].

The role of reactive oxygen species generated in the system, such as SO_4_·^−^ and HO·, was investigated by Real et al. [36], who reported second-order rate constants for the reaction between sulfate radicals and AMT as determined with competition kinetics: (4.8 ± 0.6) × 10^9^ M^−1^ s^−1^ (Figure 23) [36]. The experimental conditions started from initial concentrations [S_2_O_8_^2−^]_0_ = 50 μmol/L, [AMT]_0_ = 1 μmol/L at t = 20 °C and a neutral pH (Figure 23a). The second-order reaction was confirmed by running kinetic tests under specific initial conditions [AMT]_0_ = [isoproturon]_0_ = 1 μmol/L and [^t^BuOH]_0_ = 0.01 mol/L (Figure 23b).

**Figure 24:**
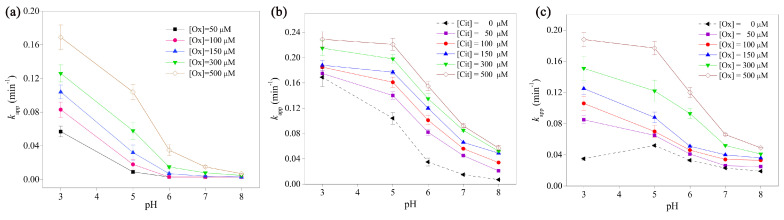
The photodegradation rate constant of AMT (**a**) in Fe(III)-Ox solutions at different initial concentrations of oxalate [Ox] and [H^+^] and (**b**) at different initial concentrations of citrate [Cit] and (**c**) oxalate [Ox]. Reprinted with permission from Ref. [92].

**Figure 25:**
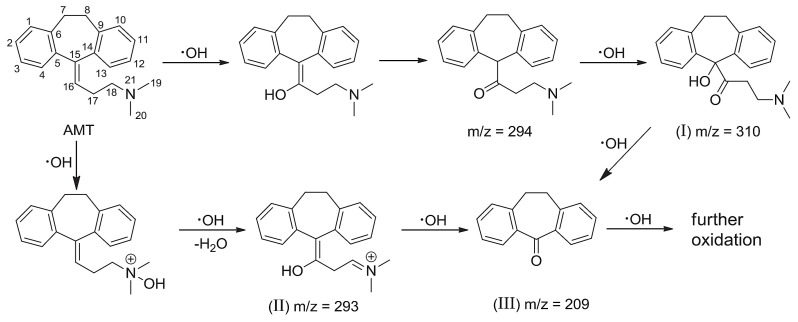
Possible photodegradation pathway of AMT in the Fe(III)-citrate-oxalate catalytic system. Reprinted with permission from Ref. [92].

A synergistic effect was observed when amitriptyline (AMT) underwent photodegradation using a catalyst based on the Fe(III)-citrate-oxalate binary system (Figure 24 and Figure 25) [92]. The photodegradation rate constant of AMT in Fe(III)-Ox solutions was assessed at different oxalate concentrations and pH levels. Typical initial conditions were [AMT]_0_ = 5 mM and [Fe(III)]_0_ = 10 mM, with variations in the citrate ([Cit]_0_ = 150 µM) or oxalate [Ox]_0_ = 500 µM concentrations (Figure 24).

Many Fe(III)-carboxylate systems have been used to date, and Fe(III)-oxalate and Fe(III)-citrate systems have been employed for the production of hydroxyl radicals and the consequent elimination of organic contaminants (Figure 25) [92]. Interestingly, the photodegradation of AMT in the Fe(III)-Cit-Ox system occurs faster than in either the Fe(III)-citrate or Fe(III)-oxalate systems, corroborating the idea of a synergistic effect in the Fe(III)-citrate-oxalate solution, which also increases the concentration of reactive species (Figure 25). 

**Figure 26:**
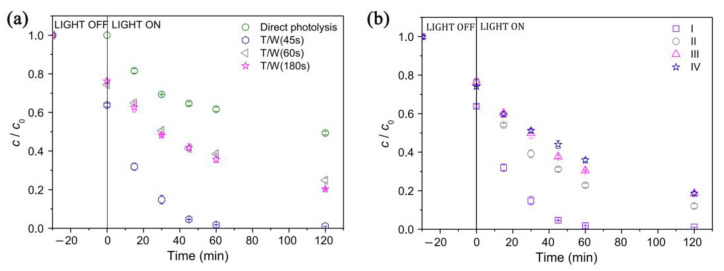
(**a**) UV-driven photolytic and photocatalytic degradation of AMI (0.03 mM) using TiO_2_/WO_3_ coatings. (**b**) Reusability of the T/W coating (45 s) in the removal of AMI (0.03 mM) using UV radiation. Reprinted with permission from Ref. [110].

Other photocatalysts have also been used for the photodegradation of AMT, such as TiO_2_ and TiO_2_/WO_3_ coatings obtained from pure titanium substrates by using plasma electrolytic oxidation. Starting from a concentration of [AMT]_0_ = 0.03 mmol/L, the activity of TiO_2_/WO_3_ coating was still high enough even after four cycles (Figure 26) [110].

**Figure 27:**
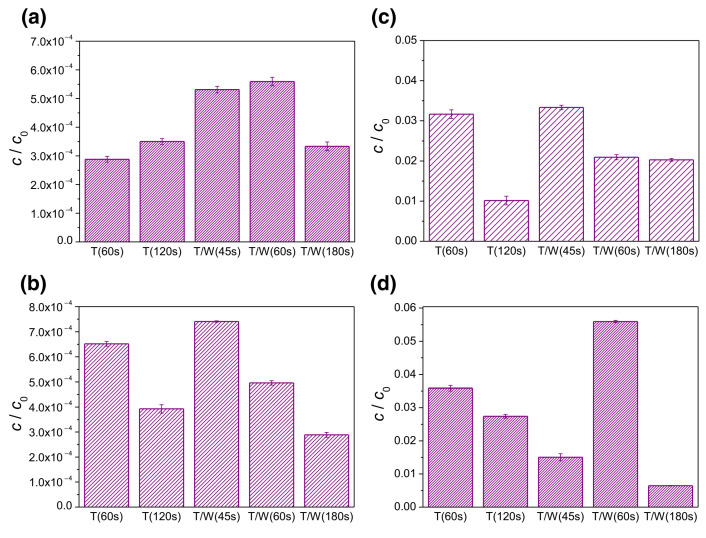
Ionic degradation products of AMI (0.03 mmol/L) formed using different coatings after 120 min of UV irradiation: (**a**) acetate; (**b**) formate; (**c**) nitrite; (**d**) nitrate. Reprinted with permission from Ref. [110].

Fincur et al. showed that the degradation of AMI produced only ionic/inorganic species, such as nitrite, nitrate, ammonium ion, acetate, and formate, as final products (Figure 27). The highest degree of mineralization (38.8%) was afforded by a T/W coating (45 s) after 120 min of irradiation. Notably, the solution obtained after irradiation was benign to the environment, as there was no toxicity in four mammalian cell lines that were tested: rat hepatoma, mouse neuroblastoma, human colon adenocarcinoma, and human fetal lung [110].

**Figure 28:**
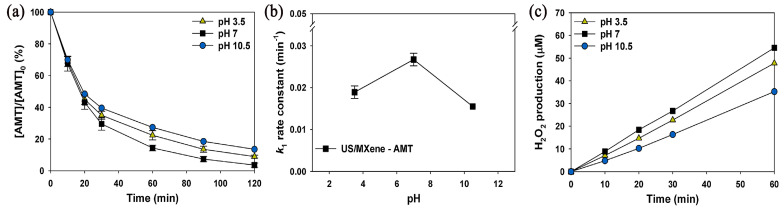
Influence of pH on the sonodegradation performance of (**a**) AMT; (**b**) degradation rate and (**c**) H_2_O_2_ production at different pH levels. Reprinted with permission from Ref. [111].

Other catalysts, such as 2D materials of the MXene type, have also been investigated for the degradation of pharmaceutical active components (PACs) and, more specifically, TCAs such as AMT [111]. It has been shown that a combination of MXene (Ti_3_C_2_T_x_) and ultrasonication can enhance the performance of AMT removal from an aqueous solution (Figure 28). The effects of a hydroxyl radical promoter (H_2_O_2_) and scavenger (^t^BuOH) were also studied in an UltraSonication reactor US/MXene system operating at 35 kHz, 50 W/L, and 293 ± 1 K [111].

**Figure 29:**
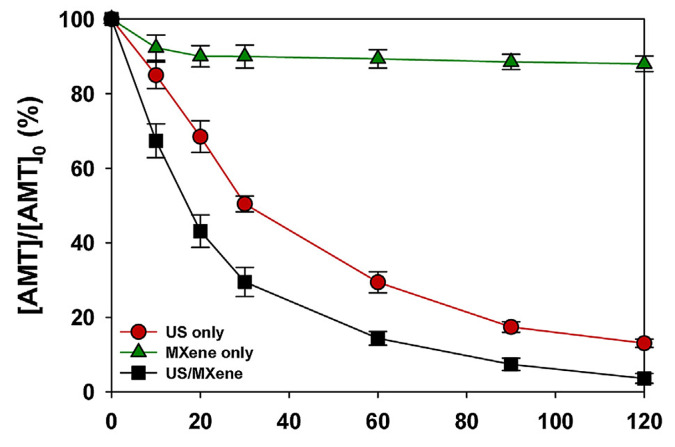
Degradation performance during an MXene-only, US-only, and combined US/MXene process for AMT. Reprinted with permission from Ref. [111].

Additionally, a combined treatment comprising of sonication and MXene exposure afforded the highest degradation rate under similar operating conditions and temperature (35 kHz, 50 W/L, pH 7, 293 ± 1 K), again pointing to synergistic effects during the investigated timeframe of 120 min (Figure 29) [111].

These case studies illustrate the importance of stability testing and understanding the degradation behavior of antidepressant drugs. They highlight the potential vulnerability of these medications to degradation under different storage conditions, pH variations, exposure to light or traces of transition-metal-based impurities such as metal salts, and the challenges associated with combination therapies. While being thermally stable beyond 100 °C, the degradation reactions usually accelerate with the increase in temperature; hence, the temperature during storage and transportation must be carefully checked. The findings emphasize the necessity of implementing appropriate storage conditions, packaging strategies, and formulation optimizations to maintain the stability, efficacy, and safety of antidepressant medications throughout their shelf life. The sheer number and findings of presented case studies contribute to a better understanding of the complexity of antidepressant drug degradation, covering areas such as pediatric formulations, manufacturing processes, and environmental impacts. The main oxidation products vary with the catalyst used, temperature, oxidative reagent, and initial concentration of reagents, and they usually consist of consecutive individual oxidation steps, where hydroxylation seems to be a prevalent mechanism. Only strong oxidants afford the final mineralization of AMT. Each case study highlights specific challenges and considerations related to the stability of AMT, providing valuable insights for pharmaceutical manufacturers, healthcare professionals, and regulatory bodies in ensuring the stability and efficacy of antidepressant medications.

## 4. Future Perspectives and Challenges

**Figure 30:**
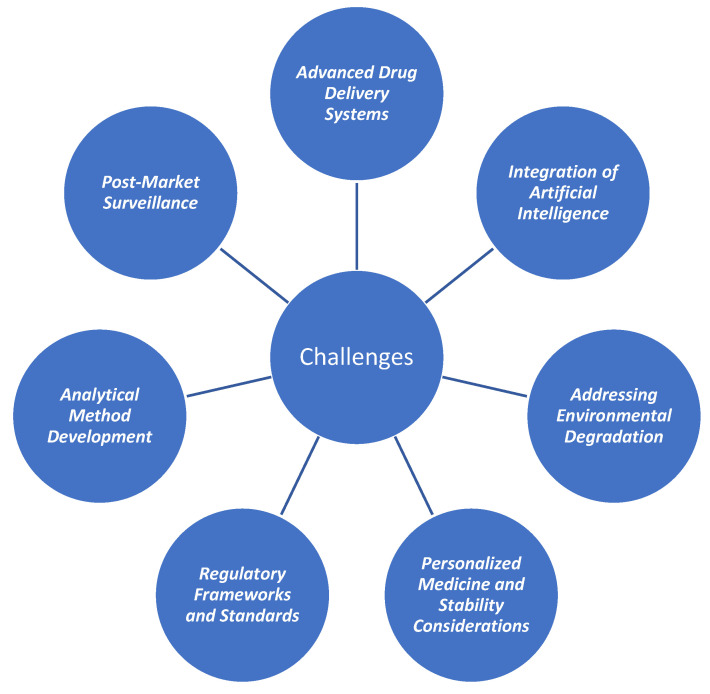
Current and future trends and challenges for antidepressant drugs.

As the demand for effective mental health treatments continues to rise, it is essential to address the stability and degradation issues associated with antidepressant medications. There are several potential improvement strategies that can ease the safe and efficient disposal of antidepressant drugs, including but not limited to advanced drug delivery systems and formulation technologies, personalized medicine, stability considerations, addressing environmental degradation, regulatory frameworks and standards, integration of artificial intelligence, the development of analytical methods, the detection of degradants, post-market surveillance, and long-term stability. The most effective approach should involve devising an integrated plan based on the key points detailed below (Figure 30).

Future perspectives in the field of antidepressant drug degradation hold great potential for advancing the stability-enhancing strategies, efficacy, and safety of these medications. Advances in formulation technologies, green chemistry, personalized medicine, and quality control practices offer promising avenues for addressing stability concerns. However, several challenges need to be addressed, including the scalability of advanced technologies, regulatory harmonization, and long-term stability monitoring. By tackling these challenges through collaborative efforts among researchers, pharmaceutical companies, regulatory agencies, and healthcare professionals, antidepressant drugs can maintain their stability, efficacy, and safety, benefiting individuals in need of mental health treatment while following a clear degradation pathway that is monitored to guarantee minimal environmental impact and pollution. Efficient degradation pathways encompass hydrolysis and forced hydrolysis at extreme pH values, thermal degradation, oxidation, and photodegradation, and these necessitate a comprehensive strategy for enhancing drug stability. Complete knowledge of medication schemes with careful consideration of potential drug–drug interactions while implementing protective measures and analytical techniques for the detection of even minute amounts of degradants can be an effective tool for healthcare professionals to mitigate degradation risks [73,77].

Forced degradation studies are instrumental in comprehending how antidepressant drugs behave during water remediation and purification processes. As these drugs increasingly find their way into water sources, concerns arise regarding their environmental impact. Through controlled forced degradation, researchers simulate environmental stressors, unraveling transformation products and degradation kinetics [73]. These insights are vital for developing water treatment strategies, identifying potential byproducts, and designing advanced purification methods. In the pursuit of water resource safety and sustainability, forced degradation studies remain a critical tool. The degradation of antidepressant drugs demands continual research and innovation to uphold stability, efficacy, and safety [39,76]. The global harmonization of stability guidelines emerges as a pivotal challenge that necessitates collaborative efforts among regulatory bodies.

The emergence of complex drug products and combination therapies presents another challenge in terms of stability, demanding a focus on compatibility studies for multi-component formulations, post-market surveillance, and interaction studies employing analytical techniques [4,75]. Ensuring long-term stability and conducting post-market surveillance are also vital considerations [40]. Robust pharmacovigilance programs must be established to monitor stability issues beyond the approval stage and ensure the continuous assessment of long-term stability and potential degradation risks. On the same note, the formation and persistence of toxic transformation products should be thoroughly evaluated while considering their potentially adverse consequences for the ecosystem.

The future of antidepressant drug stability lies in integrating innovative technologies, sustainable practices, and personalized medicine. Overcoming regulatory challenges, addressing complexities in drug formulations, and advancing analytical techniques require collaborative efforts. This unified approach promises improved patient outcomes, enhanced mental health treatment, and readily available and stable antidepressant medications.

## Figures and Tables

**Table 1:** Degradation of typical TCA drugs and their decomposition products.

Antidepressant	Chemical Formula	Degradation Type and Products; Observations
**Amineptine**	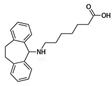	Produces *N*-dealkylated metabolite, Nortriptyline. Acts as a DRI (dopamine reuptake inhibitor) and weak norepinephrine reuptake inhibitor.
**Amitriptyline**	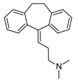	Produces Nortriptyline as a metabolite, a potent antidepressant, and (*E*)-10-hydroxynortriptyline. Inhibits serotonin transporter (SERT) and norepinephrine transporter (NET).
**Amoxapine**	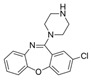	Identified degradation products: [2-(2-aminophenoxy)-5-chlorophenyl]-piperazin-1-yl-methanone and 2-chlorodibenzo[b,f][1,4]oxazepin-11(10*H*)-one [94]. The N-demethylated metabolite of loxapine. Decreases the reuptake of norepinephrine and serotonin (5-HT).
**Clomipramine**	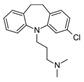	Photodegradation products: imipramine, HO-imipramine, desmethylclomipramine, and HO-imipramine-N-oxide [98]. Other authors have identified the photodegradation of CMP to produce only OH-imipramine (*m*/*z* = 297.2) [93]. Selective serotonin reuptake inhibitor (SSRI).
**Desipramine**	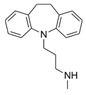	There are 3 main photolysis routes for desipramine (DES): isomerization, hydroxylation, and ring opening [84]. Oxidation occurs through the loss of the 1-methylaminopropyl moiety; 18 decomposition products could be envisioned [84]. Selective norepinephrine reuptake inhibitor; weak serotonin reuptake inhibitor.
**Doxepin**	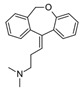	UV photodegradation includes E/Z isomerization of the heteroaryl-conjugated alkene group of doxepin [2]. Two identified decomposition products: OH-doxepin and doxepin N-oxide [98]. Enhances the neurotransmitter’s serotonin (5-HT) and [norepinephrine (NE)]_brain_.
**Imipramine**	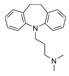	Iminodibenzyl and desimipramine are the major degradation products (AIBN stress testing) [90]. UV radiation from simulated sunlight produced demethylation and hydroxylation of imipramine [101]. Acts as a strong SSRI (serotonin reuptake inhibitor)
**Maprotiline**	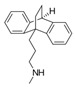	Degradation to 12 products through hydroxylation/oxidation and ring opening. Acts as a strong inhibitor of the histamine H_1_ receptor (sedative) or through the inhibition of presynaptic uptake of catecholamines (treatment of depression, or anxiety).
**Nortriptyline**	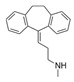	The main metabolite is 10-E–hydroxynortriptyline [37]. NTRI, an active metabolite of amitriptyline, acts by inhibiting the reuptake of the neurotransmitter serotonin and is used for neuropathic pain, attention deficit hyperactivity disorder (ADHD), and anxiety.
**Opipramol**	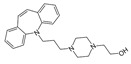	The main degradation product is (10-3-[4-(2-hydroxyethyl)-piperazinyl]propyl]acridine) [106]. Acts as a SIGMAR1 agonist.
**Trimipramine**	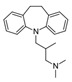	Desmethyltrimipramine, 2-hydroxytrimipramine, and trimipramine-N-oxide are metabolites of trimipramine. TMP decreases the reuptake of norepinephrine and serotonin (5-HT) to treat major depressive disorder or insomnia.

**Table 2:** A summary of representative degradation strategies employed to degrade AMT.

System	Degradation Type	Detection Method	Catalyst/Oxidant/Degradation Agent	Ref.
AMTH/CoFe-LDH/CF	Heterogeneous electro-Fenton system	UPLC-QTOF-MS; ICP–MS (Co, Fe)	Layered double-hydroxide structure (LDH): CoFe-LDH/CF cathode	[47]
AMT/Ru(III)/KMnO_4_/H^+^	Chemical oxidation	UV-Vis	Active catalyst [Ru(H_2_O)_6_]^3+^	[108]
AMT/acidic aq. FA (fluvic acid)/HO^−^	AOP (demethylation, hydroxylation)	MS	FA* (excited FA triplet states)	[53]
AMT/FeOCl	Chemical degradation/mineralization	HPLC	Catalyst: iron oxychloride (FeOCl)/peroxymonosulfate (PMS)	[52]
AMI (amitriptyline hydrochloride)/ZnO	Photocatalytic degradation	UV-Vis	Catalyst: ZnO; reactive radical species: [HO·]	[50]
AMT/H^+^ or HO^−^	Forced hydrolysis	HPLC, MS	H^+^/HO^−^/[O] (when H_2_O_2_ was used)	[87]
AMT (solid state)	Thermal degradation	ATR–FTIR	– (ICTAC 2000 protocol)	[11]
AMT/H_2_O_2_	AOP	GC-MS, HPLC	HO· derived from H_2_O_2_	[51]
AMT/hν (sunlight)	Photodegradation	LC–MS	–	[93]
AMT/Co-TNW	UV-Vis exposure; hydroxylation	MS	cobalt-titanate nanowires (Co-TNW)	[16,109]
AMT/artificial gastric juice	Forced hydrolysis	LC/UV	H^+^ (pH =1.2); no degradation observed	[94]
AMT/S_2_O_8_^2−^	UV/S_2_O_8_^2−^ oxidation	HPLC	SO_4_^−^ and HO·	[36]
AMT/Fe(III)	Photodegradation	MS	Fe(III)-citrate-oxalate	[92]
AMT/TiO_2_/WO_3_	Photocatalytic	LC, UV-Vis	TiO_2_ and TiO_2_/WO_3_ coatings	[110]
AMT/MXene	Sonodegradation under UV	HPLC	Ti_3_C_2_T_x_ MXene, H_2_O_2_ (HO· promoter), ^t^BuOH (radical scavenger)	[111]

## Data Availability

No new experimental data were created in this study. Data sharing is not applicable to this review article.
